# Nessys: A new set of tools for the automated detection of nuclei within intact tissues and dense 3D cultures

**DOI:** 10.1371/journal.pbio.3000388

**Published:** 2019-08-09

**Authors:** Guillaume Blin, Daina Sadurska, Rosa Portero Migueles, Naiming Chen, Julia A. Watson, Sally Lowell

**Affiliations:** MRC Centre for Regenerative Medicine, Institute for Stem Cell Research, School of Biological Sciences, University of Edinburgh, Edinburgh, United Kingdom; University of Dundee, UNITED KINGDOM

## Abstract

Methods for measuring the properties of individual cells within their native 3D environment will enable a deeper understanding of embryonic development, tissue regeneration, and tumorigenesis. However, current methods for segmenting nuclei in 3D tissues are not designed for situations in which nuclei are densely packed, nonspherical, or heterogeneous in shape, size, or texture, all of which are true of many embryonic and adult tissue types as well as in many cases for cells differentiating in culture. Here, we overcome this bottleneck by devising a novel method based on labelling the nuclear envelope (NE) and automatically distinguishing individual nuclei using a tree-structured ridge-tracing method followed by shape ranking according to a trained classifier. The method is fast and makes it possible to process images that are larger than the computer’s memory. We consistently obtain accurate segmentation rates of >90%, even for challenging images such as mid-gestation embryos or 3D cultures. We provide a 3D editor and inspector for the manual curation of the segmentation results as well as a program to assess the accuracy of the segmentation. We have also generated a live reporter of the NE that can be used to track live cells in 3 dimensions over time. We use this to monitor the history of cell interactions and occurrences of neighbour exchange within cultures of pluripotent cells during differentiation. We provide these tools in an open-access user-friendly format.

## Abstract 2

A new computational method allows researchers to measure the properties of individual nuclei in situations in which cells are tightly packed together, for example, during differentiation of stem cells in culture or during early postimplantation development.

## Introduction

Studying the properties of individual cells in relation to their neighbours in intact tissues is the first step towards understanding the cell–cell interactions that govern the behaviour of tissues. Single-cell analysis can also reveal heterogeneity in cellular properties that is masked by lower-resolution population-averaging methods. Automated computational image analysis is a particularly attractive approach because it is free from operator bias, provides quantitative data, and reveals subvisual information that would not otherwise be apparent [1–4].

In order to meet the need for automated nuclear segmentation in 3D, a wide array of methods have been developed, reviewed in [2,5–7]. It is becoming increasingly apparent, however, that there is no single ‘one size fits all’ solution to segmentation. New solutions are needed for situations in which nuclei are densely packed, nonspherical, or heterogeneous in shape, size, or texture: these things apply to many embryonic and adult tissue types as well as to cells differentiating in culture. There is also a need to reduce the time and computational power required for segmentation of each cell: this becomes a limiting factor when imaging whole embryos or large tissues, when analysing time-lapse data, or in any other situation in which large numbers of nuclei need to be identified. Finally, adoption of published methods by the community can be limited, highlighting the importance of creating well-documented and user-friendly software [4,8,9].

Here, we report a new approach to overcome these bottlenecks in quantitative image analysis of individual cells in 3D. Rather than relying on staining for nuclear content (for example, DAPI or Hoescht staining), we instead detect the nuclear envelope (NE). This makes it easier to identify individual nuclei that are in close contact with each other and does not suffer from segmentation problems associated with textured nuclear staining, unusually shaped nuclei, or cell debris. Furthermore, the NEs of individual nuclei are easily discernible by eye in crowded tissues, so manual correction of any mis-segmented nuclei becomes easier than is the case for DAPI-stained nuclei. We provide a user-friendly 3D–4D editing tool to rapidly correct any segmentation errors.

We test our method alongside other previously published user-friendly methods, with focus on mid-gestation embryos, 3D cultures of pluripotent stem cells, and pluripotent stem cell–derived neural rosettes. These contexts are chosen because they exemplify many of the problems in 3D segmentation that we set out to address: crowded overlapping nuclear signals, nonspherical nuclei, large numbers of nuclei, and decrease in signal intensity towards the centre of large structures. Using our editing tool, we manually annotated thousands of nuclei in these contexts to generate a set of segmented images, which are made publicly available and which could be useful as a standard segmentation benchmarking dataset [10].

We consistently obtain accurate segmentation rates of >90% in these challenging contexts. We demonstrate the utility of this tool by measuring the expression of transcription factor 15 (Tcf15) at single-cell resolution across the anterior–posterior (AP) axis of the embryonic day (E)8.75 mouse embryo. We also developed a nondisruptive method to fluorescently label the NE in live cells and used this to follow the history of cell–cell interactions and to document neighbour exchange as pluripotent cells differentiate in 3D culture.

This new tool, named ‘Nuclear Envelope Segmentation System (Nessys)’, adds to the expanding toolkit of segmentation approaches, each of which has its particular strengths. Nessys is particularly well suited for segmenting large numbers of nuclei arranged in complex 3D configuration without requiring extensive amounts of time, computational power, or user input. We provide Nessys as an open-access, well-documented, easy-to-install, and user-friendly software.

## Results

### Segmentation of nuclei in groups of densely packed cells

**Fig 1:**
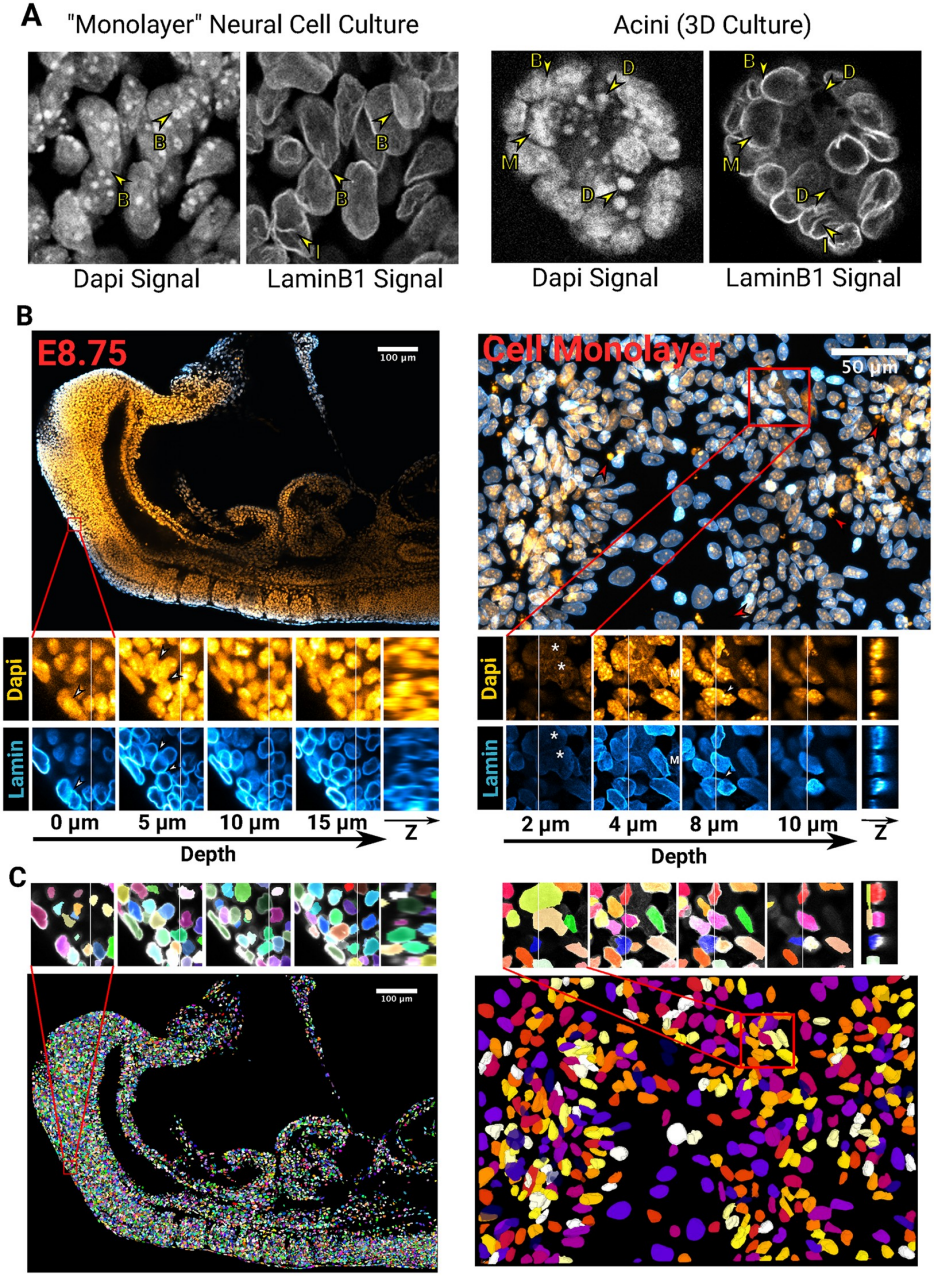
Segmentation challenges and Nessys results. (A) Side-by-side comparison of the DAPI and LaminB1 signals obtained by confocal microscopy of pluripotent stem cells differentiated into neural cells (left) or grown in a 3D matrix (right). ‘B’ arrows indicate boundaries between touching nuclei, ‘D’ arrows point at cell debris, the ‘M’ arrow shows a mitotic nucleus, and ‘I’ arrows indicate invaginations of LaminB1 into the nucleus. (B) Confocal micrographs showing the diversity of cell shapes and volumes found in an E8.75 mouse embryo (left) and within a ‘monolayer’ of cultured cells (right). DAPI is shown in orange and LaminB1 in cyan. For each image, a magnified region is shown as a series of planes along the *z* axis of the image for both the DAPI channel or the LaminB1 channel. An image constructed along the *yz* axes is also shown. The faint vertical bar in the *xy* planes indicate the location of the *yz* image. White arrows: loss of nuclei edge in the DAPI signal, red arrow: cell debris apparent in the DAPI signal, white asterisks: flat and large nuclei distinct from their surrounding cells. (C) Images of segmented nuclei obtained with Nessys. Nuclei are assigned a unique label and a random colour. The same regions as in (B) are shown. Notice how overlapping nuclei with distinct morphology are identified accurately. E, embryonic day; Nessys, Nuclear Envelope Segmentation System.

Nuclei are commonly detected based on fluorescent markers of nuclear content, for example, DAPI. However, it can become difficult to distinguish individual cells based on DAPI staining when nuclei are densely crowded, for example, during neural differentiation of pluripotent cells (Fig 1A left) or 3D cultures (Fig 1A right) or in densely packed tissues in vivo (Fig 1B).

We decided to avoid this problem by using the NE as a landmark for segmentation. Staining for LaminB1, an intermediate filament that marks the NE [11], allows crowded nuclei to be clearly distinguished by eye (Fig 1). This observation prompted us to explore whether using the NE signal as an input for an automated detection method would be a successful approach.

This led us to develop a method called Nessys that is able to reliably detect individual nuclei in crowded groups (Fig 1C, S1 Movie). In the following section, we give an overview of this method and a demonstration of its utility.

### Overview of the Nessys segmentation method

**Fig 2:**
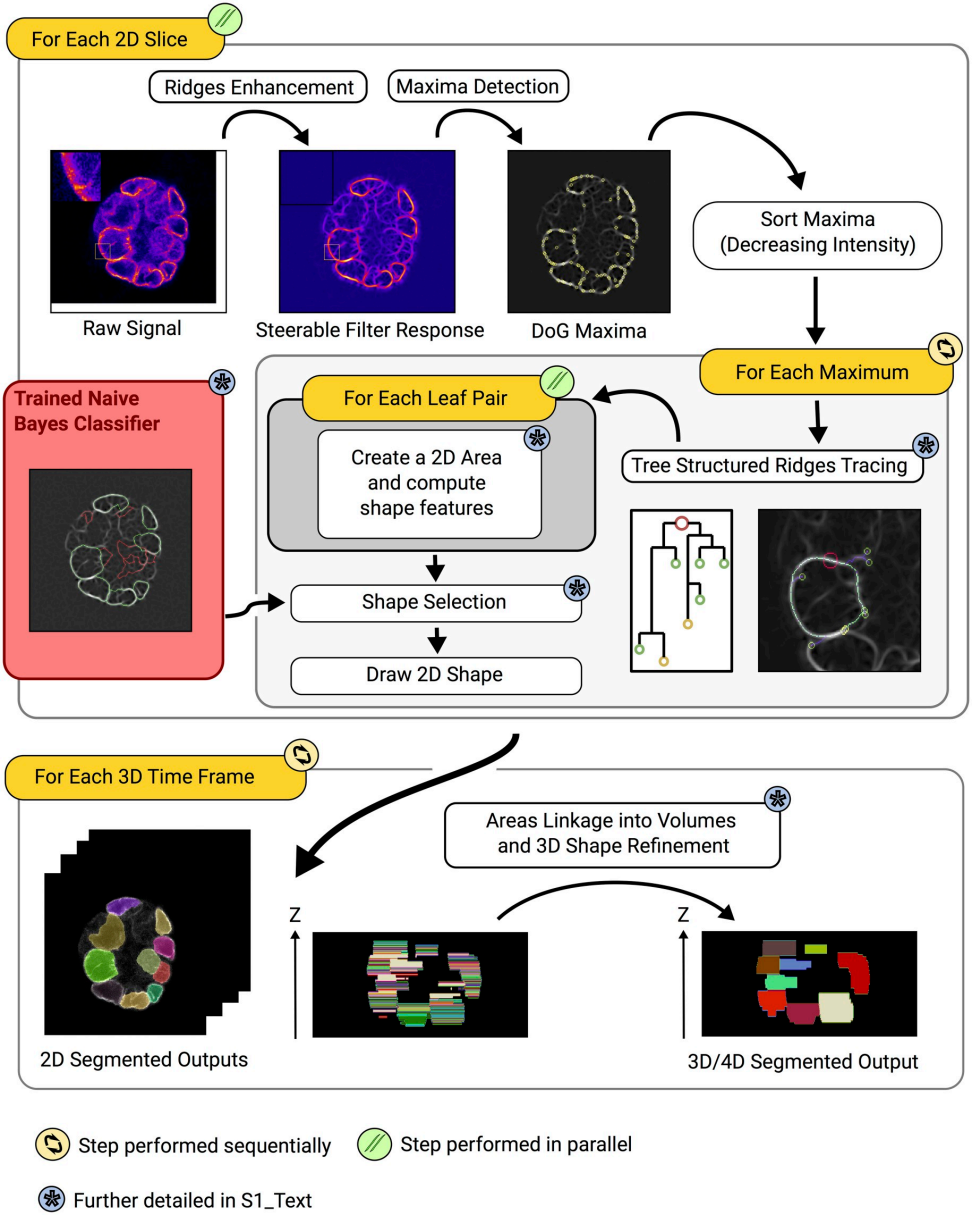
Overview of the Nessys segmentation method. The main steps of the method are shown in white boxes annotated with a blue asterisk whenever the step is further detailed in S1 Text. Where possible, a snapshot of the intermediate output is shown. Iterations are represented with orange boxes annotated with an icon to indicate whether iterations are parallelised or sequential. Instructions that are part of the same iteration are contained within the same rounded box. In the ‘tree-structured ridge-tracing’ step, a full tree is overlaid on the image and corresponds to the diagram on the left of the image. The red circle represents the root (maximum where the procedure was initialised), smaller circles indicate the leaves of the tree, and lines represent the branches of the tree. The use of a (reusable) trained naive Bayes classifier is shown with a red box. This classifier is trained by the user before running the method. DoG, Difference of Gaussian; Nessys, Nuclear Envelope Segmentation System.

The main steps of the Nessys segmentation method are briefly summarised here (Fig 2). An in-depth description of complex procedures (asterisks in Fig 2) is also available in S1 Text section A.

- The NE signal is first subjected to a ridge detector (steerable filter) developed by [12]. This filter enhances and smoothens the ridge regions. This improves the ridge-tracing step by making it easier to accurately follow the contour of nuclei.
- Regions of maximum fluorescence intensity are then identified using a Difference of Gaussian detector [13]. These form the starting points for a dynamic ridge-tracing process, which continuously identifies and moves to the brightest adjacent pixel in an iterative process (see also S1 Fig and S2 Movie).
- Over time, this tracking algorithm identifies branch points and iteratively establishes the most likely segmentation solution using a naive Bayes classifier trained by the user (S1 and S2 Figs, S1 Text section A2).
- Once this process has been completed for each *z* slice, 2D areas are linked together into 3D volumes. At this point, information from adjacent slices helps refining the initial 2D segmentation results (S3 Fig, S1 Text section A3).

In this method, the 2 most computer-intensive tasks are the ridge-tracing procedure and the optional volume-refinement procedure (S3F Fig). To reduce computational time, 2D slices and 3D volumes are processed in parallel for each procedure respectively.

### An open-access utility program and dataset for the benchmarking of nuclear segmentation methods

Determining the performance of a segmentation method requires a gold standard (or ground truth [GT]) image dataset [10,14–16]. GT datasets may be computer generated [17–19] or may consist of images that have been manually segmented by biologist specialists [15]. Recent initiatives have undertaken to make such datasets publicly available [10]. However, currently available GT datasets for nuclear segmentation only contain a ‘nuclear content’ signal and, in most cases, do not encompass the diversity in nuclear shape and sizes or the level of complexity found in mammalian systems.

**Fig 3:**
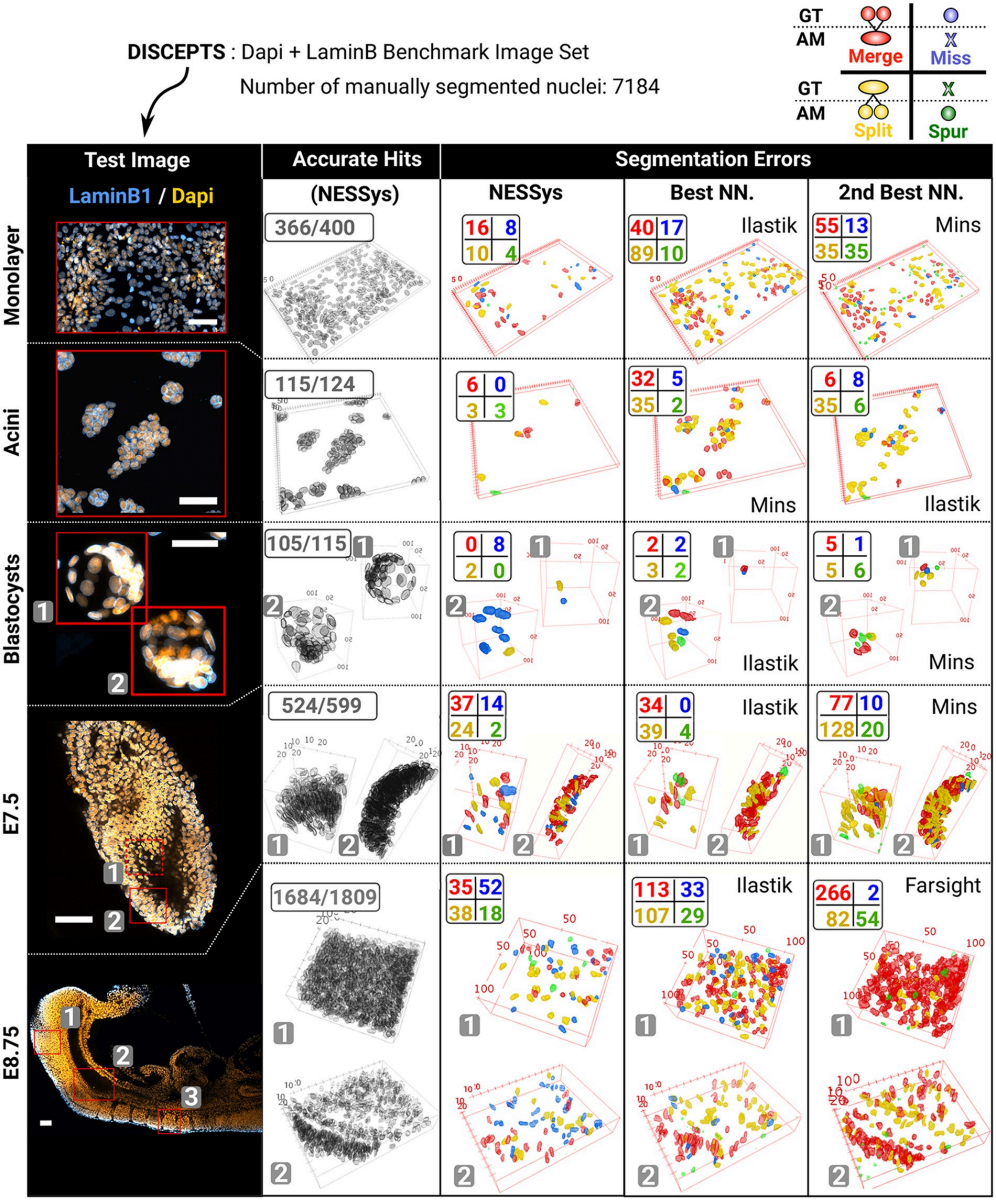
Overview of the manually segmented image dataset and sample error maps created with Nessys validation and benchmarking tools. Representative test images of the DISCEPTS image set are shown in the left column of the table (scale bars: 50 μm). For each image, red outlines represent the manually segmented image regions used to evaluate accurate hits and errors, which are shown as 3D maps in the other columns. If multiple regions are drawn, a number in a grey box indicates the correspondence between a given region and the matching 3D map. Second column: Accurate nuclei identified with Nessys. Third column: Maps of Nessys errors. Fourth and fifth column: Map of errors for the best and second-best NN. methods. The legend for these errors is indicated in the top right corner of the figure. Merge: Only one nucleus found in AM when several nuclei are present in GT. Miss: Nucleus found in GT but absent from AM. Split: Several nuclei found in AM when only one is present in GT. Spur: Nucleus generated by AM that does not exist in GT. Please note that the ‘Monolayer’ and the ‘E8.75’ images are the same images as in Fig 1B. AM, automated method; E, embryonic day; GT, ground truth; Nessys, Nuclear Envelope Segmentation System; NN., non-Nessys; Spur, Spurious.

For these reasons, we have generated a new image dataset with thousands of manually annotated nuclei for segmentation benchmarking. We used both an NE signal (LaminB1) and the more conventional chromatin signal (DAPI) (Fig 3 and S1 Table). Images include:

- High-density monolayer culture of differentiating mouse embryonic stem (ES) cells
- 3D cultures of differentiating mouse ES cells embedded in Matrigel
- E3.5 mouse blastocysts
- Gastrulating mouse embryo at E7.5, focusing on a region of the distal epiblast
- Postgastrulation mouse embryo at E8.75, focusing on 3 regions of the embryo that capture both epithelial and mesenchymal structures as well as variations in signal intensity because of light scattering or antibody penetration issues

We called this dataset DISCEPTS (‘Differentiating Stem Cells & Embryos are a Pain To Segment’), as images contain crowded, overlapping, heterogeneous, nonspherical nuclei in several different contexts, representing the particular segmentation challenges that we have set out to address. DISCEPTS was deposited to the Image Data Resource [20] under accession number idr0062 (https://idr.openmicroscopy.org). To enable independent validation of our results, we also provide all Nessys variables used to segment the DISCEPTS dataset in S2 Table and training sets for the classifiers as supplementary data (S1 Data).

In order to perform both manual annotation of nuclei in 3D and segmentation benchmarking, we developed additional programs that we provide alongside the Nessys method (S4 Fig):

- A 3D painting tool to establish GT segmentation data (we estimate that approximately 100 3D nuclei were manually segmented per hour). Note that this tool can also be used as a visual inspector and 3D editor to curate outputs from any automated methods.
- An extensible segmentation comparator that can compute the benchmarking metrics described in [15] as well as 3D maps of accurate hits and segmentation errors (Fig 3 and S4 Fig and S3 Table). This makes it possible to visually compare the frequency and distribution of each class of segmentation error for each tested segmentation output.

Using these new tools and our DISCEPTS dataset, we compared the performance of Nessys to other previously published popular methods listed below:

- MINS [21]: originally designed for detecting cells within preimplantation embryos based on nuclear content
- Farsight [22]: designed for detecting nuclei based on nuclear content
- Ilastik [23]: a more generalist method that uses machine learning (to harness the full Ilastik capability and obtain the best performance, we used both the NE and the DAPI channels as input to inform the Ilastik method; see also Materials and methods).

The table of 3D error maps shown in Fig 3 hinted at the fact that Nessys does perform particularly well in regions where nuclei are densely packed or where the cells are flat and overlapping (monolayer and acini). We did notice a slightly higher proportion of missed nuclei in embryos compared to non-Nessys methods. When inspecting individual images, we were able to confirm that this issue could mainly be attributed to the loss of NE signal when the cells undergo mitosis. Overall, this preliminary analysis indicated that Nessys performs comparably with other tools for relatively simple tasks (e.g., blastocysts) and may have an advantage in more challenging contexts.

### Quantification of performance metrics of Nessys in comparison with other methods using the DISCEPTS dataset

**Fig 4:**
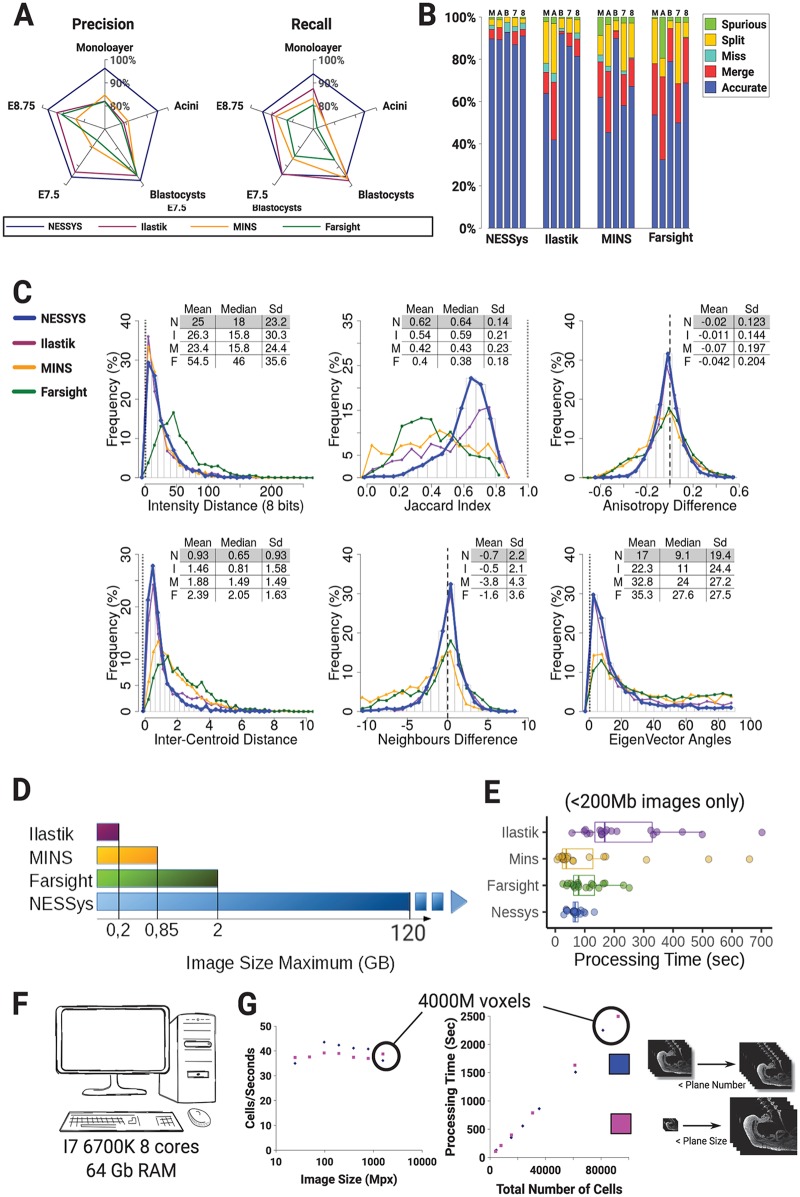
Error counting, morphological accuracy, and computational time benchmarking. (A) Radar charts representing precision and recall for each tested method over the DISCEPTS dataset. (B) Stacked bar chart showing the proportion of detection accuracy and errors for each tested segmentation method (‘M’: Monolayer, ‘A’: Acini, ‘B’: Blastocysts, ‘7’: E7.5, ‘8’: E8.75). (C) Histograms of the deviation of morphological features from the ground truth (to create a single graph for each feature, 200 cells from each biological dataset were randomly sampled and pooled together so each dataset would contribute equally to the graph). A table summarises the mean and SD of the distribution for each tested method (‘N’: Nessys, ‘I’: Ilastik, ‘M’: MINS, ‘F’: Farsight). The vertical dashed line shows the value of the measure for the ground truth (see also S1 Text section B3). (D) Maximum image size successfully processed by the tested methods in GB. Dashes indicate that the largest tested image was successfully processed. (E) Processing times in seconds recorded for each tested method on <200-Mb benchmarking images. (F) Specifications of the computer used for benchmarking. (G) Scatter plot of the number of processed cells per seconds by Nessys versus the size of input image (E8.75 zone 1 resized by varying either plane sizes or number of planes while keeping resolution identical). The same input variables were used throughout. Data tables listing individual measurements used for the figure are available on GitLab (https://framagit.org/pickcellslab/data/2019_nessys) except for panel (G), which are given in S5 Table. E, embryonic day; GB, gigabytes; Mpx, megapixels; Nessys, Nuclear Envelope Segmentation System; RAM, random-access memory.

To further assess the performance of the tested methods on the DISCEPTS dataset, we represented precision and recall for each method and biological specimen as a radar plot (Fig 4A). Precision measures the proportion of the shapes in the tested image that correspond to actual shapes in the GT image (manually segmented image). Recall indicates the proportion of shapes in the GT image that are present in the tested image. In other words, precision is a measure of how much of the detected shapes are indeed real, and recall measures how much of the real shapes have been detected (see also S1 Text section B 1 and 2). The F-measure, which combines precision and recall, is given in S4 Table.

We confirm that all methods perform well for segmenting nuclei in blastocysts, in which nuclei are generally near spherical in the densest region (inner cell mass [ICM]) or well separated (trophectoderm [TE]). However, Nessys achieved close to or above 95% precision and recall where other methods failed to achieve satisfactory results (Fig 4A). In particular, the greatest discrepancy was observed for images of cells grown in culture (monolayer and acini), in which the cells are often flat, overlapping, and very heterogeneous in shape.

The summary of error counts shown in (Fig 4B) confirms this analysis. Importantly, the balanced proportions of under- and oversegmentation events and the minimal proportion of missed or spurious events provided a good indication that variables were properly adjusted for all methods (by ‘spurious’, we mean nuclei generated by the automated method, which do not exist in GT).

Ultimately, the purpose of automated nuclear segmentation is to fulfil the need for the quantification of biologically relevant features. Therefore, we then assessed the deviation of automated methods from GT in the distribution of generated features related to shape, intensity, or neighbourhood (Fig 4C).

One of the most frequent application of automated segmentation in biology is its use in generating fluorescence-activated cell sorting (FACS)-like profiling of gene expression from immunofluorescence data, i.e., quantitative immunofluorescence [24–29]. To test performance on this aspect, we simulated the signal from 3 heterogeneously expressed transcription factors and computed a distance from GT intensities (see S1 Text section B3). We observed that Nessys, Ilastik, and MINS performed similarly with a median of the distribution of distance under 20 arbitrary fluorescence units (AFU). This was an interesting observation indicating that intensity measurement was relatively robust to segmentation errors—notably, MINS error counts were greater in all biological samples and the Jaccard Index (JI) deviation from GT (Fig 4C), whereas Nessys’ JI was the closest from GT.

Nuclear shape features are particularly relevant when studying morphogenesis [2,30–35]. For this reason, we computed the shape’s anisotropy and the angle between the main axis of the best-fit ellipsoid for a detected shape and its matching GT shape. In this case, segmentation errors were much less forgiving; only Nessys and Ilastik provided satisfactory results; and when looking at results for individual biological samples, these features deviated from GT for Ilastik when Ilastik segmentation resulted in the greatest number of errors (Monolayer and acini, S5 Fig).

We also assessed performance on the detection of the number of neighbours, as this feature can be particularly relevant when studying collective organisation of the cells in various contexts [36–41]. Again, only Ilastik and Nessys resulted in an accurate neighbour count with a standard deviation of 2.2 neighbours from GT. This could be explained by the particular ability of Nessys and Ilastik to identify precisely the position of the shapes centroid and to result in shapes with higher JI than other methods (neighbourhood identification uses both centroids and the shape of the nuclei; see Fig 4C bottom right image and section B.3 in S1 Text).

Altogether, our data provide an overview of the level of accuracy that can be expected from Nessys segmentation on biologically relevant features, and we conclude that Nessys is a satisfactory method for applications involving measuring intensities, anisotropy, polarity, and neighbour analysis.

Finally, we addressed the problem of processing time becoming limiting for large images. Using a desktop computer with 64 GB of random-access memory (RAM), no method other than Nessys was able to process images larger than 2 GB in our hands, whereas Nessys was able to process a time-lapse image dataset of more than 120 GB. This property of Nessys was enabled by the underlying SCIFIO I/O library [42] and ImgLib2 image structure [43], which made it possible to process each time frame sequentially and which handled larger than memory images. Processing limitations depend on the computing resources available, but Nessys has the advantage of making it feasible to use a typical lab computer to segment large images.

Some of the methods we tested failed to complete segmentation (Fig 4D) or resulted in prohibitively long segmentation times when images were larger than 200 MB; we therefore report processing times for non-Nessys methods for image crops < 200 MB only (Fig 4E and S6 Table). Although in some cases processing time was shorter than with Nessys, Nessys was on average faster than other methods and never exceeded a processing time of 132 s, whereas maximum processing time was found to be 251, 660, and 702 s for Ilastik, Farsight, and MINS, respectively (Fig 4E).

We noticed that with other methods, processing time increased proportionately with image size, which was not the case with Nessys (S6 Table). To investigate this aspect, and as Nessys was capable of segmenting large images, we artificially varied image size of the E8.75 image by either varying the size or the number of image planes. We observed that the time taken to segment each nucleus remained constant regardless of image size with an average of 40 nuclei per second for the set of variables used in this experiment (Fig 4F and 4G). In other words, Nessys processing time increases linearly with the number of nuclei in the image and not with image size. This property can be explained by the fact that Nessys only uses 2 image filters, which need to work on every voxel of the image, and these filters work in parallel on individual 2D planes. Subsequent steps work on detected maxima, whose quantity is proportional to the number of nuclei in the image.

Overall, we conclude that Nessys performs particularly well for large images and 3D images containing crowded nuclei and provides a useful addition to the toolkit of segmentation approaches currently available.

### Impact of image quality on Nessys accuracy

Next, we decided to document how image quality might impact Nessys accuracy (S6–S8 Figs).

By design, Nessys can accommodate a wide range of nuclei intensities as demonstrated by Nessys segmentation in the E8.75 image, in which a strong drop in fluorescence intensity is visible in the deeper layers of the tissue (Fig 1 and S6A Fig). Nevertheless, in order to estimate the signal-to-noise ratio that would lead Nessys to fail, we gradually increased the amount of (Gaussian) noise and resegmented the image using the same variables throughout (S6B Fig). We observed only little influence of noise on accuracy until noise reached a standard deviation of 300, and then accuracy started to decrease significantly (S6C and S6D Fig). However, we could rescue accuracy in the noisiest image by adding 5 ‘noisy shapes’ to the training set of the classifier and applying image smoothing included as an optional step in Nessys (S6C and S6D Fig and S1 Text section C.1).

Next, we set out to determine how the sampling rate in the *z* axis might affect Nessys accuracy. To do so, we resampled the E8.75 zone 1 image to create a series of images with *z*-step sizes ranging from 0.5 μm to 5 μm. We then segmented all images using the same variables except for the min and max volume, which we adjusted accordingly (S1 Text section C.2).

We found that in this particular case, accuracy started to drop with *z*-step size above 1.2 μm (corresponding to an average of approximately 6 optical slices per nucleus). This result shows that Nessys is not particularly robust to large *z*-step sizes. However, this limit is reasonable, since this resolution is routinely achievable with a standard confocal microscope. This result also illustrates how the 3D linkage step captures 3D information to correct for 2D segmentation errors.

Finally, we tested the effect of bit depth on Nessys results. We converted 2 of our 12-bit images into 8-bit images and resegmented the images with Nessys (S8 Fig and S1 Text section C.3). To our surprise, bit depth had no effect on Nessys results whatsoever. Importantly, we used the same classifier for both 12-bit and 8-bit images. The only variable that needed modification was the threshold of the maxima detection task. If downstream applications do not necessitate a 12-bit intensity range, this can be a great advantage to reduce image file size.

Altogether, our data show that Nessys is robust to noise and that it can be used on samples with wide variations in nuclei intensities and with image resolutions that are routinely achievable with standard laboratory imaging equipment.

### Measurement of gene expression and morphological features in postimplantation embryos

**Fig 5:**
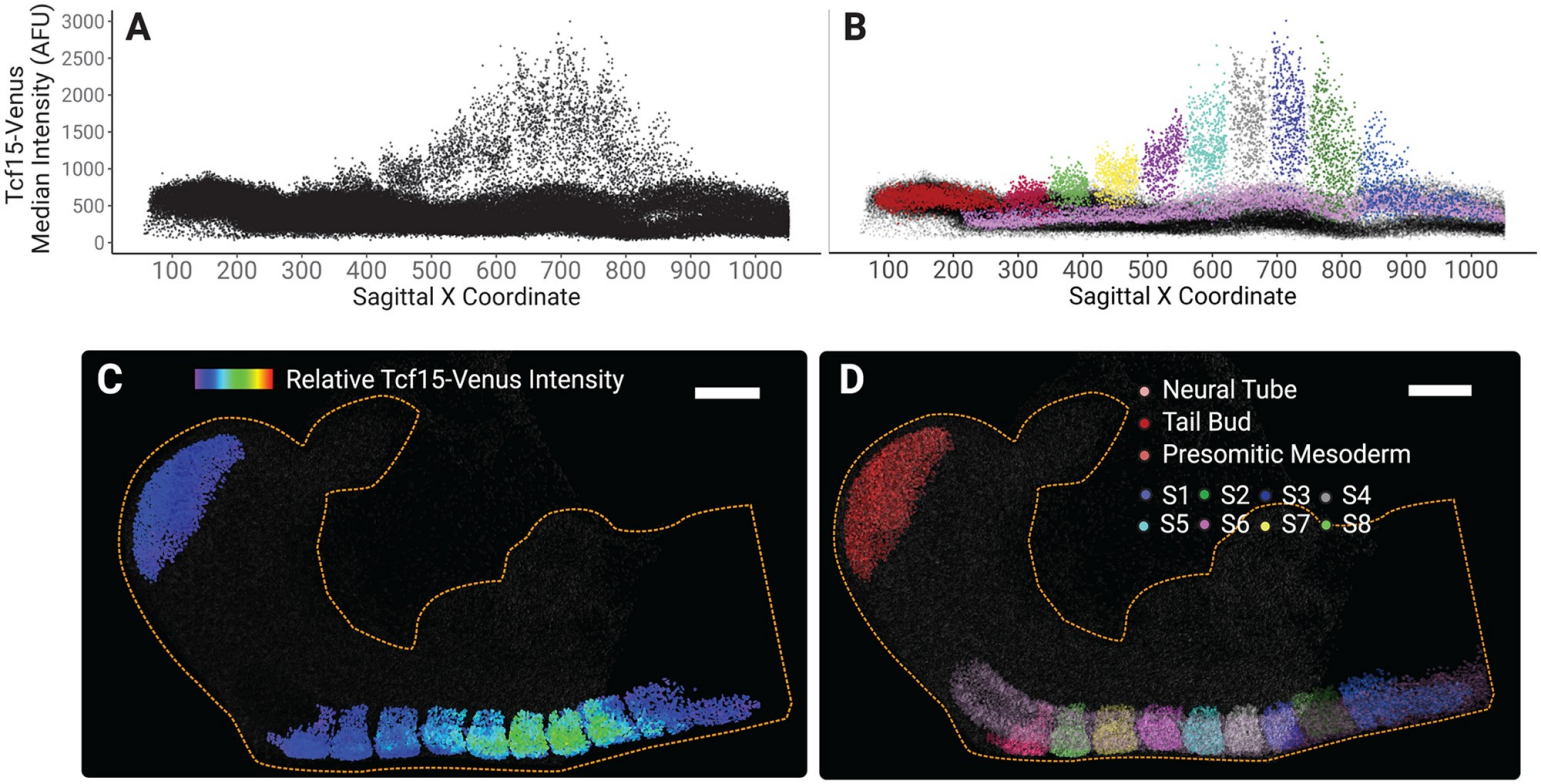
Quantitative analysis of Tcf15 expression in a complex E8.75 mouse embryo. (A) Scatterplot of the median Tcf15-Venus intensity within the cell versus the cell position along the Sagittal X coordinate. Boundaries between somites are apparent as gaps between groups of Tcf15-high cells. (B) Same as (A) except that points are colour-coded according to their respective embryonic region as shown in (D). (C) 3D rendering of the all detected nuclei in the E8.75 mouse embryo image. The heatmap of the relative Tcf15-venus intensity is shown only for the tailbud and somitic regions; other regions are shown in grey. (D) 3D rendering of nuclei grouped by embryonic regions. Scale bar in (C) and (D): 100 μm. The yellow dashed outline delineates the shape of the embryo. Data tables listing individual measurements used for the figure are available on GitLab (https://framagit.org/pickcellslab/data/2019_nessys). AFU, arbitrary fluorescence units; E, embryonic day; Tcf15, transcription factor 15.

To illustrate the usefulness of Nessys segmentation and the associated 3D painter for the study of large embryos, we made use of mouse embryos that we have engineered to express Venus under the control of the regulatory elements of Tcf15, a transcription factor that marks the formation of somites [44]. The signal of the Tcf15-Venus reporter is included in the E8.75 image of the DISCEPTS dataset. Using Nessys segmentation on the full image, we generated a plot of the median Tcf15 intensity per nucleus versus position of nuclei along the AP axis of the embryo (Fig 5A). We observed that within the fraction of cells that expressed higher levels of Tcf15 than average (>1,000 AFU), several clusters of points along the AP axis were clearly apparent. Using the Nessys 3D painter to manually annotate individual somites as well as other anatomical landmarks (Fig 5B, 5C and 5D), we confirmed that these point clusters reflected spatial organisation of the somites.

Tcf15 was known to be expressed in somites, but no analysis at single-cell resolution has previously been reported. We observe a gradual increase in Tcf15-Venus fluorescence as somites age, reaching a peak at the seventh oldest somite (S3), after which expression declines sharply in the oldest somites S1–2 (Fig 5A, 5B and 5C). Notably, low levels of expression were also detected within the presomitic mesoderm prior to formation of the first somite. We also observe that Tcf15-Venus expression displays considerable heterogeneity within each somite.

This analysis illustrates how the combination of Nessys segmentation and 3D annotation tool can be used to quantify large multidimensional images of complex embryos with good accuracy and within a reasonable time frame.

### A fluorescent NE reporter for the automated tracking of live cells

Next, we set out to label the NE in live cells in order to leverage Nessys segmentation power as an input for cell tracking.

Whereas live markers of the whole nucleus are well characterised (for example, H2B–fluorescent protein [FP] [45] and FP–nuclear localisation signal [NLS] [46] are commonly used for cell tracking [10,14,21,33,47–49]), this is not the case for NE markers.

**Fig 6:**
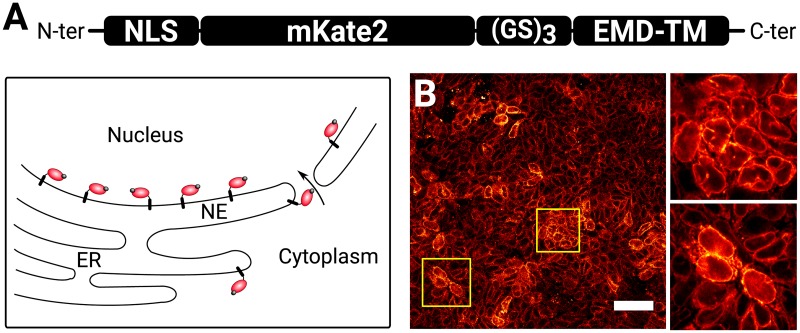
A novel synthetic NE fluorescent protein for live cell tracking. (A) Schematic representing the structure of the NE live reporter and its expected topology and localisation within the cells. (B) High-resolution confocal image showing the reporter localisation within a stable cell line constitutively expressing the reporter. EMD-TM, 44 C-ter amino acids from the human Emerin protein (UniprotKB: P50402) that contain a transmembrane domain; ER, endoplasmic reticulum; (GS)_3_, Gly-Ser linker; NE, nuclear envelope; NLS, nuclear localisation signal.

An ideal live reporter needs to be bright, accurately localised, and nondisruptive to cell function when constitutively expressed. Lamins are well known to regulate cell function, and even small changes in their sequence or their expression levels can have deleterious effects [11,50]. Similarly, fluorescent tagging of other NE-associated proteins may disrupt the function of the NE and affect cell function [51]. Given these considerations, we generated NE-mKate2, a chimeric construct consisting of the bright mKate2 FP [52] linked to an NLS and attached to the single-pass transmembrane domain of Emerin (EMD) [53]. We reasoned that this construct would be constantly imported into the nucleus while being tethered to the intramembranar network of the cells (Fig 6). Indeed, random integration of NE-mKate2 driven by a CAGS promoter [54] into mouse ES cells resulted in a bright signal with a robust localisation to the nuclear rim (Fig 6). As this construct is devoid of any known protein interaction motif apart from the NLS, we expect this protein to be inert to cell function. Notably, we confirmed that NE-mKate2 ES cells were able to contribute at high efficiency to chimeric embryos, indicating that the fluorescent label had no obvious deleterious effect on the cells [55]. To conclude, the NE-mKate2 construct is a novel nondisruptive fluorescent reporter for detecting the NE in live cells.

### Tracking cell–cell interactions during differentiation of pluripotent cells

**Fig 7:**
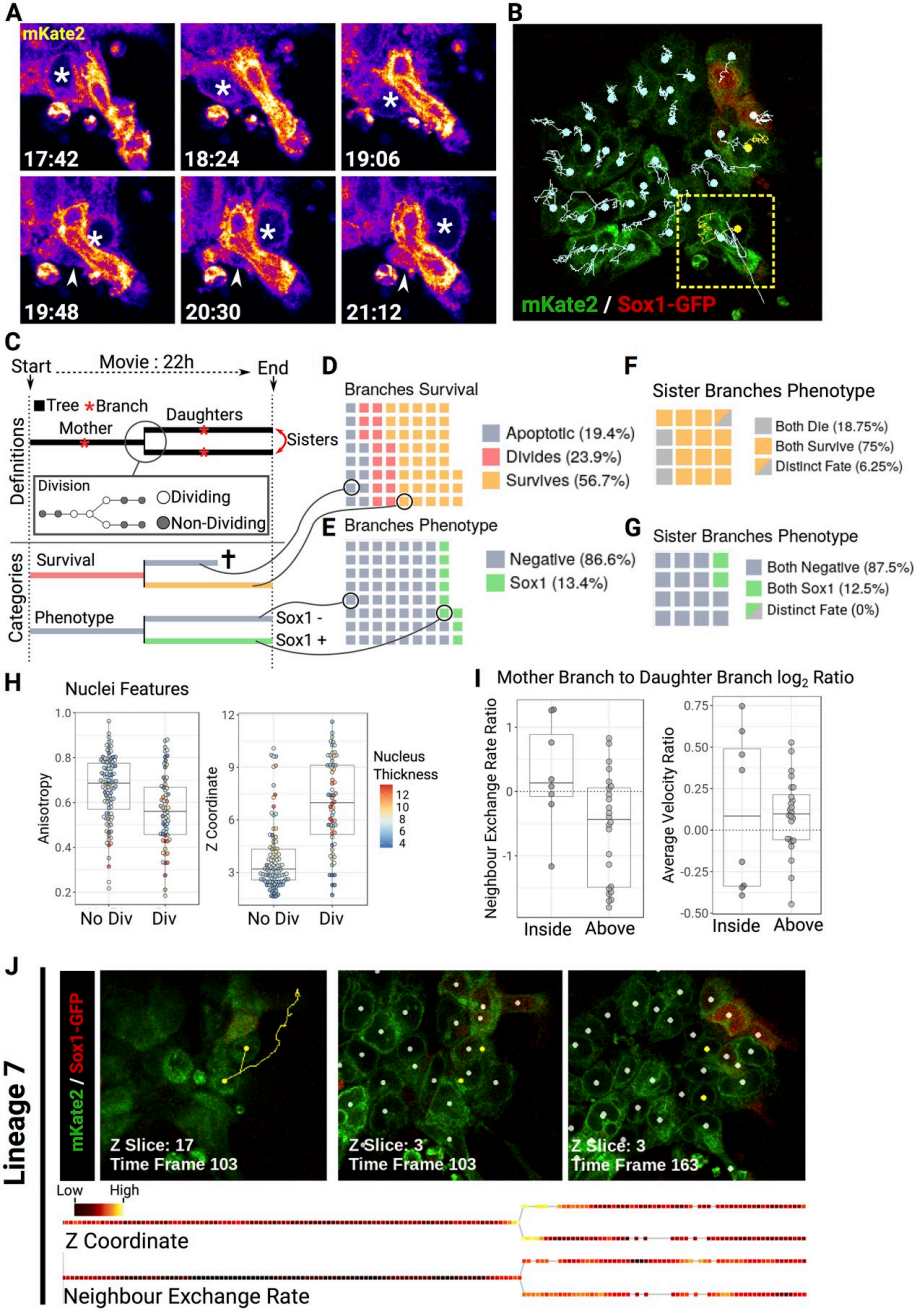
Lineage and neighbour exchange analyses reveal that cell mixing is favoured by divisions above the epithelial plane. (A) Magnified snapshots from a single optical *z* slice of S3 Movie. An asterisk indicates a cell moving underneath a bright elongated cell. The arrowhead shows the trailing side of the moving cell. Time is indicated as HH:MM. (B) Single frame from the Sox1 time-lapse experiment. Individual tracks are overlaid on top of the mKate2 (green) and Sox1 (red) signals. The dots indicate the current position of the detected cells. Location of the snapshots shown in (A) is indicated by a yellow square. (C) Schematic representing the different part of a lineage tree and the colour code used for classification. (D-E) Waffle charts representing the destiny of individual lineage branch either in terms of apoptosis, survival, or division (D) or in terms of Sox1 identity (E). Each square represents one branch in the dataset. (F-G) Waffle charts comparing the destiny of sister branches for survival (F) or for differentiation (G). (H) Beeswarm-boxplots showing the *z* coordinate or the anisotropy of dividing and nondividing nuclei. (I) Beeswarm-boxplots showing the log_2_ ratio of mother branch to daughter branches of the average velocity or neighbour exchange rate. (J) Representative track (yellow) containing a division above the epithelial plane, which leads to greater neighbour exchange rate after division (white dots represent other detected cells in the image). The corresponding colour-coded trees based on *z* coordinate or neighbour exchange rate are shown below images (gaps in the tree correspond to time frames within which the cell was not detected). Note the high *z* coordinate at division and the increase in neighbour exchange rate after division. Data tables listing individual measurements used for the figure are available on GitLab (https://framagit.org/pickcellslab/data/2019_nessys). GFP, green fluorescent protein; Sox1, SRY-box transcription factor 1.

To explore the utility of our tools for identifying cells within time-lapse datasets, we performed confocal live imaging of pluripotent cells differentiating into neural cells. We used mouse ES cells containing an SRY-box transcription factor 1 (Sox1)–green fluorescent protein (GFP) reporter [56] in order to detect Sox1+ neural progenitors as they emerge over time (Fig 7A, S3 Movie). We chose this system because during the first few days of this process, flat cells form tight 3D clusters in which it becomes difficult to distinguish individual cells (Fig 7A). Notably, the neural monolayer culture condition was amongst the most challenging dataset to segment according to our benchmarking results (Fig 4A and 4B).

Although excellent tools exist for the automated live tracking of 2D cultures [37,57–60], the situation described here would not be possible to address with these tools because of the propensity of differentiating mouse ES cells to squeeze underneath each other (Fig 7A and S3 Movie), making it necessary to perform analysis in 3 dimensions.

One possible approach to tackle this challenging situation would be to perform mosaic labelling to make it easier to track a few fluorescent cells among predominantly unlabelled cells. This would be sufficient to extract a subset of cell lineages but would fail to capture local cell–cell interactions or the broader multicellular context surrounding each cell, both of which are central to understanding the emergent properties of differentiating populations [37,41]. For this reason, we decided to label all the Sox1-GFP cells with our NE-mKate2 construct in order to analyse the collective behaviours of differentiating cells in 3D.

Unlike snapshot images, in which Nessys segmented cells with high accuracy without user intervention, tracking of this challenging time-lapse image required a significant amount of manual correction. However, Nessys segmentation plus manual correction could be completed comfortably within 1 d (approximately 7 h), whereas manual segmentation of the entire dataset would have taken around 70 h (220 time points with an average of 30 cells per frame and at a rate of 100 cells segmented per hour). After segmenting each individual frame, we ran a basic tracker (S1 Text section E.2) and were able to obtain accurate lineage trees (Fig 7B and 7C, S3 Movie).

We first tracked rates of cell death, cell survival, cell division (Fig 7D), and differentiation (Fig 7E). Interestingly, when we compared the destiny of sister cells, we observed that the majority of sister pairs underwent the same fate in terms of survival (Fig 7F) and that all underwent the same fate in terms of differentiation (Fig 7G). This observation is consistent with the idea that differentiation decisions can be made several generations before reporters of cell fate become detectable [61,62].

We next monitored incidents of neighbour exchange (S1 Text section E2.1), making use of the ability of Nessys and associated tools to track the position of cells in 3D. Neighbour exchange is thought to occur frequently in the mouse epiblast, but it is not known to what extent this process influences the distribution of differentiating cells in culture.

We hypothesised that a major contributor to neighbour exchange would be the position of divisions with respect to the plane of cell growth. Indeed, when we explored the 3D organisation of the cells over time, we noticed that the *z* coordinate of the cells increased when cells were dividing (anisotropy was decreasing slightly as expected; however, the change in *z* coordinate was uncorrelated to the change in cell thickness, Fig 7H). This raised the possibility that as cells divide above other cells, daughter cells would reintegrate into the colony at different locations, thus acquiring new neighbours. To measure this, we set a *z*-coordinate threshold above which divisions were considered to be occurring above the plane, and we classified lineages accordingly (S1 Text section E2.2). Then, we compared the neighbour exchange rate occurring before division and after division, and we observed that when the cells divided above the plane (which accounted for 33% of the total number of divisions), neighbour exchange rate was more likely to increase, thus supporting our hypothesis (Fig 7I). Notably, we confirmed that this observation was unlikely to be explained by a change in cell velocity (Fig 7I right). A division representative of this phenomenon is shown in Fig 7J.

Although this dataset is too small to draw definitive biological conclusions, it illustrates that in principle, it is possible to use Nessys and associated tools to measure the position of each cell in 3 dimensions and assess cellular behaviours of these cells with respect to their neighbours over time, even in challenging contexts when individual cells are tightly packed and difficult to distinguish by eye.

## Discussion

In this study, we set out to explore the idea of using the NE for nuclear segmentation as an alternative to DNA/chromatin labelling. This idea was proposed more than 15 y ago, and preliminary results on small 2D images were promising [63]. Surprisingly, however, this idea had not yet, to our knowledge, been revisited for complex 3D datasets. Here, we present Nessys, a method tailored to automatically segment nuclei on the basis of the NE. We show that this method performs particularly well for ‘difficult’ datasets, in which images contain a large array of nuclear shapes and sizes and nuclei overlap. It is also a method that is fast and scales well to large images. We discuss next the strengths and weaknesses of our method, and we summarise our effort to make the method easy to adopt by the community and the usefulness of the tools and dataset we provide with this article.

### Features of the NE signal explain Nessys performance

To construct the DISCEPTS dataset, we focused on sampling a diversity of nuclei shape, size, texture, and 3D tissue organisation. The images we gathered provide a good illustration of the multiple challenges that still need to be addressed in nuclear segmentation, and we believe this dataset will be useful as a GT dataset for benchmarking purposes.

Importantly, this dataset illustrates that different cell types harbour different organisation of their chromatin, leading to very heterogeneous appearances of the nuclear content. For example, the nuclei of neural cells contain regions of high affinity for DAPI that can be seemingly randomly distributed within the nucleus, including at the periphery. This property can confound methods relying on the assumption that brighter regions reside at the centre of the shape. Also, nuclei boundaries that are key features for many methods [2,5–7] become very difficult to distinguish even by eye. These problems are avoided when using an NE staining, and this explains in part the success of the Nessys method. Nuclear boundaries remain clear when NE-stained nuclei are in close contact with one another (Fig 1B), and indeed, our results show that the two methods that make use of the NE signal (Nessys and Ilastik) performed particularly well for all embryo images (Fig 4A and 4B, S4 Table).

One potential disadvantage of the NE signal is that the interior of the nucleus may become difficult to differentiate from the exterior of the nucleus. As this distinction is easily achievable by the human eye, Nessys uses a naive Bayes classifier trained on a small subset of shapes in order to exclude nonrelevant shapes (internuclear spaces). This classification ensures that only one channel is required to achieve proper nuclear segmentation and leaves other colours available for other purposes. This contrasts with Ilastik, which in our analysis required the DAPI channel to perform well.

Our results also revealed that the biggest difference with other tested methods was observed for images of cells grown in culture. Although mammalian embryos are recognised to be amongst the most challenging systems to segment [14,33,48], we show that differentiating stem cells in culture can be equally if not more difficult to segment accurately, as they tend to overlap [64] and be flatter and less regular in shape than their in vivo counterparts. Because of the drop in resolution in the *z* axis and in NE signal at the top and bottom of the nuclei, even Ilastik failed to separate overlapping cells (Fig 4A and 4B). Nessys performs well, as it makes full use of the optimal *xy* resolution first and then uses a graph-colouring strategy to link 2D shapes into 3D volumes.

We would like to emphasise that different methods are appropriate for different contexts and that our method is intended to complement, rather than replace, existing methods, being particularly well suited for segmenting images with complex 3D arrangements of irregularly shaped nuclei.

Finally, to complete the documentation of Nessys capabilities, we also tested Nessys ability to segment other types of signals commonly used in 2D high-content screening or 3D analysis (S9 Fig and S1 Text section D). Our results show that although Nessys was not designed to segment DAPI-stained nuclei, membrane-labelled cells, or differential interference contrast (DIC) images, Nessys can still provide accurate segmentations in those situations. This demonstrates that our approach has potential to be optimised for these types of signals as well.

### Adoptability of the Nessys method

#### Practicality of using NE over nuclear content

DNA-labelling dyes are easy to use and, for this reason, are widely adopted to mark the nuclei of the cells. Such dyes do not yet exist for the NE, so usage of Nessys requires that the NE be stained with antibodies. This may potentially impose constraints on applications because of host-species restrictions. We used LaminB1, as it is known to be expressed in most cell types with minimal cell–cell variability [65], but other NE epitopes could be used: we provide in S7 Table alternative antibodies that provide similar signals in our hands. The use of nanobodies might further improve practicality, reduce antibody penetration issues, and be more animal friendly, although we have not tested this approach ourselves [66].

We have also developed a NE live reporter for the use of Nessys for time-lapse imaging (Figs 6 and 7). Given that several simple approaches are available for labelling the NE, we anticipate that most users should be able to find a method suitable for their own system.

#### Software usability

The community is in need of open-source, well-documented, and easy-to-use software in order to accelerate quantitative, reproducible, and collaborative research [4,8,9]. In order to align with this goal and to facilitate adoptability of Nessys, the software is free and open-source (https://framagit.org/pickcellslab/nessys), and the installation process only takes a couple of minutes and does not require particular knowledge in computing. Nessys has been tested on Linux, Windows, and MacOS.

A video tutorial and documentation for Nessys are available online, and the user interface includes interactive tools for aiding variable adjustments (see also the issue tracker, which allows comments from the community). The software and associated documentation will continue to be improved in response to user feedback. A longer-term goal is to remove the need for variable adjustments altogether. Notably, we envision the possibility of using a few annotated volumes with the Nessys editor as a training set for automated variable adjustments.

An important practical aspect of Nessys is that it is designed to be fast and to avoid problems due to processing times per cell becoming limiting. We have shown that Nessys can produce accurate segmentation results in a reasonable time frame even for large datasets on a standard lab workstation (Fig 4).

#### Code portability, extensibility, and future work

We developed Nessys using good software design practice. For instance, the user interface uses a modular wizard pattern so that individual subtasks may be improved without modifying others. This will facilitate future improvements of the method. Such improvements may include replacing the last morphological filtering step with a modern shape-refining method [67] or using more sophisticated classifiers [68] during the shape-ranking process to further improve accuracy. Future work will also focus on enhancing visualisation of segmentation quality in order to speed up the segmentation editing process, for example, by including a colour map of the segmentation confidence score as described in [69].

Nessys has initially been developed as a module for our own framework but is designed to be portable to other general purpose frameworks such as ImageJ [70] or Icy [71].

This will help bridge our method with other powerful image analysis components. For example, using an image restoration method such as CARE [72] upstream of Nessys segmentation together with downstream sophisticated tracking methods [10,49] will bring us a step closer to the goal of recording and interrogating the emergent properties of collective organisation of the cells in context.

In conclusion, we hope that the tools presented here will contribute to accelerating quantitative research. We provide a robust 3D nuclear segmentation method together with a 3D editor. The software is easy to install as a stand-alone application and is designed to be portable to general purpose frameworks such as ImageJ. We also provide a dataset and a utility program for segmentation benchmarking in the hope that they will be useful to the community. These tools are free and open-source and designed to work on a standard lab computer with datasets generated from conventional microscopes.

## Materials and methods

### Ethics statement

Animal experiments were performed under the UK Home Office project license PEEC9E359, approved by the Animal Welfare and Ethical Review Panel of the University of Edinburgh and within the conditions of the Animals (Scientific Procedures) Act 1986.

### Software development and availability

Nessys and associated programs are written in Java. Our programs only depend on open-source libraries. These include ImgLib2 [43] for the image data structure and SCIFIO [42] and BioFormats [73] for image input/output. We developed our code in Eclipse March 0.3 (https://www.eclipse.org/). Dependencies are managed using Maven and version control with Git. Source code is available on GitLab at Framasoft (https://framagit.org/pickcellslab/nessys).

Implementation details, source code, and user documentation are subject to changes as we continue to improve the software. Source code and updated documentation may be found on our GitLab repository hosted by Framasoft (https://framagit.org/pickcellslab/nessys). We created a Git tag ‘v0.1.0’ to label the commit that corresponds to the version of the software that was used for this publication.

### ES cell culture

All mouse ES cell lines were routinely maintained on gelatinised (Gelatin, Sigma) culture vessels (Corning) in Glasgow Minimum Essential Medium (GMEM, Sigma) supplemented with 10% foetal calf serum (FCS, APS), 100 U/ml LIF (produced in-house), 100 nM 2-mercaptoethanol (Gibco), 1X nonessential amino acids (Gibco), 2 mM L-Glutamine (Gibco), 1 mM Sodium Pyruvate (Gibco). For 2i-LIF culture [74], the cells were maintained in N2B27 medium supplemented with 3 μM of Chiron and 1 μM of PD0325911. N2B27 consists of a 1:1 ratio of DMEM/F12 and Neurobasal media supplemented with 0.5% modified N_2_, 0.5% B27, and 2-β mercaptoethanol (all from Invitrogen). Cell culture was maintained at 37 °C with 5% CO_2_ and routinely tested for mycoplasma contamination.

### Neural monolayer differentiation

Sox1-GFP ES cells [56] were first differentiated into EpiSC following the protocol described in [75] and then further differentiated into neural rosettes as follows: a confluent culture of EpiSC was diluted 1/40 and plated onto dishes coated with growth factor–reduced Matrigel (Corning) in N2B27 medium containing 10 μM of SB435215. The medium was replaced every day, and the cells were passaged every 2 d at a ratio of 1:3 to 1:6 onto Matrigel-coated dishes until neural rosettes became clearly apparent (5 d). On the last passage, the cells were replated onto 12-mm Matrigel-coated glass coverslips and fixed for immunostaining after 2 d of culture.

### Acini/3D cell culture

Wild-type E14tg2 alpha ES cells, Tcf15-Het cells, and Tcf15-KO ES cells (Lin, Tatar et al., in preparation) were used for this dataset (the name of the cell lines used for a given image is included in the name of the image). Cells were maintained in 2i-LIF condition and then plated onto Matrigel-coated dishes into N2B27/1%KSR medium supplemented with 12 ng of bFgf and 20 ng of Activin A, as described in [76], and containing 600 μg/ml of growth factor–reduced Matrigel to induce the formation of 3D structures. The cells were fixed for immunostaining on day 2 of EpiLC induction.

### Mouse strains, staging, and husbandry

Wild-type, outbred MF1 mice and transgenic mice—Tcf15-Venus were maintained on a 12-h-light/12-h-dark cycle. For timed matings, noon on the day of finding a vaginal plug was designated as E0.5. Staging of early mouse embryos was done according to [77, 78].

In order to generate Tcf15-Venus mouse lines, Tcf15-Venus ES cells were generated on an E14tg2a background by replacing the first exon of Tcf15 with the coding sequence for Venus. The targeting construct for Tcf15 contained a 2.3-kb 5′ homology arm and a 2.4-kb 3′ homology arm corresponding to the sequences flanking the first coding exon of Tcf15. Within the two homology arms is the coding sequence for Venus followed by an frt-flanked pgk-neo MC1-TK cassette selection cassette. This cassette was removed by transfection of pFlpO after isolation of successfully targeted clonal cell lines. A PGK-DTA cassette was placed upstream of the 5′ homology arm to enable negative selection of clones in which the targeting cassette had randomly integrated. Heterozygous Tcf15 ES cells were used to generate germ-line chimeric mice which were then backcrossed for several generations onto a Bl/6 background.

### Embryos collection and dissection

For blastocysts, embryos were obtained at 8-cell morula stage by washing E2.5 oviducts with M2 medium (Sigma). The zona pellucida was removed by a brief wash in Acid Tyrode’s solution (Sigma) at room temperature. Embryos were cultured in KSOM medium (LifeGlobal) at 37 °C, 5% CO_2_ for 24-h prior immunostaining. E7.5 and E8.75 embryos were isolated from the decidua in M2 medium, and the Reichert’s membrane was removed before fixation and immunostaining.

### Immunofluorescence, embryo clarification, and imaging

Samples were fixed in 4% formaldehyde/PBS/0.5% Triton X-100 for 10 min (cell cultures) or 30 min (embryos) at room temperature. For large embryos, the fixative was quenched with 100 mM Glycine/PBS for 5 min. After 3 consecutive washes with PBS/Triton, samples were incubated for a minimum of 30 min (cell culture) or overnight (embryos) with blocking solution, which consisted of 5% donkey serum (Sigma), 0.1% Triton X-100 (Sigma), and 0.03% Sodium Azide (Sigma) in PBS. Incubation with antibodies was performed for 1 h (cells) or overnight at room temperature (>E7.0 embryos) or at 4 °C (blastocysts). Antibodies were all diluted in blocking solution. Antibodies used for each image are given in S1 Table, and dilutions and references are listed in S7 Table. Samples were counterstained with 1 μg/ml DAPI (Sigma) diluted in PBS for 10 min (cells and blastocysts) or 30 min (>E7.0 embryos) at room temperature. Cells grown on glass coverslips were mounted in ProLong Gold Antifade Mountant (Molecular Probes) 24-h prior imaging. E7.5 and E8.75 embryos were further clarified using a Benzylalcohol/Benzylbenzoate (BABB)-based method adapted from [79]. Briefly, embryos were dehydrated in graded methanol series (25%, 50%, 75%, 90%, 100% twice) of 5 min each. Embryos were then transferred to a 1:1 solution of BABB/Methanol for 5 min before a final transfer into a pure solution of BABB inside a glass-bottom metallic chamber for imaging. Blastocysts were placed on a 10 μl PBS drop covered with mineral oil (Sigma) in a glass-bottom metallic chamber. Microscope and objectives used for imaging are listed in S1 Table.

### Segmentation methods: Versions, variable adjustments, and benchmarking

The 64-bit versions of Ilastik (version 1.1.9), Farsight Linux (version 0.4.4), and Nessys (v0.1.0) were tested on a desktop computer (i7 6700K 8 cores cpu, 64 GB of RAM, and a 7,200 rpm HDD) running Linux Opensuse Leap 42.3. MINS (version 1.3) was run on the same computer but on a Windows 8 OS with Matlab (version R2016a 9.0.0.341360).

In order to obtain the best segmentation output possible for each method tested, we followed recommendations available for each tool (http://katlab-tools.org/, https://github.com/RoysamLab/Farsight-toolkit, https://www.ilastik.org/documentation/index.html). For Nessys, MINS, and Farsight, we screened 5 segmentation attempts with distinct sets of variables and retained the output resulting in the best F-measure when compared to the GT (S4 Table). For Ilastik, we first trained the pixel classifier on both the LaminB1 and DAPI channels to recognise 3 pixel classes: background, nuclear content, and ‘internuclear space’ (*z* axis included), which often consisted of a bright LaminB1 signal. Using this method greatly improved Ilastik’s ability to separate touching nuclei in the subsequent steps. We then tested 5 sets of thresholding variables and retained the best segmentation result as explained previously.

Processing times were recorded by starting and stopping a digital stopwatch manually. Nessys also logged processing times to the terminal. Farsight was run from the command line while all the other methods were run from their graphic interface.

Metrics listed in S4 Table, summarised in Fig 4A and 4B, and error maps shown in Fig 3 were computed with our segmentation comparator program (S4 Fig), and morphometric measurements shown in Fig 4C and S5 Fig were computed within PickCells (https://pickcellslab.frama.io/docs/) as detailed in S1 Text.

### NE reporter construct and cell line

To generate the NE reporter, we used Gibson assembly to ligate an NLS-mKate2 fragment and the human EMD transmembrane domain (nucleotides 878–1,012 of NM_000117.2) downstream of a CAGS promoter included in a hygromycin resistance–containing backbone. mKate2-NLS was PCR amplified from pTEC20, which was a gift from Lalita Ramakrishnan ([80] Addgene plasmid #30179; http://n2t.net/addgene:30179; RRID:Addgene_30179), and the EMD sequence was PCR amplified from a full-length human EMD–containing plasmid (kind gift from Dr E. Schirmer). The NLS and (GS)_3_ linker sequences were included in the primers’ overhangs.

The resulting plasmid was validated by full sequencing of the insert and will be deposited to Addgene.

To generate the Sox1-GFP/mKate-NE double reporter cell line, we lipofected 2.5 μg of plasmid (Lipofectamin 3000, Thermofischer) according to the manufacturer’s recommendation into the Sox1-GFP cell line [54]. Hygromycin-resistant clones were selected for the best compromise between high mKate2 intensity and proper NE localisation of the transgene.

### Time-lapse imaging and tracking

For the tracking experiment, the sox1-gfp/NE-mKate2 cell line was maintained in 2i/LIF condition and then differentiated into the neural lineage as follows: The cells were dissociated with accutase (Invitrogen) and replated at a density of 10,000 cells/cm^2^ into N2B27/1%KSR medium. We determined that Sox1-GFP became expressed between day 2 and day 3 of differentiation and so replated the cells at day 2 of differentiation into phenol-free N2B27/1%KSR medium inside a glass-bottom metallic chamber for imaging. Imaging was performed with an inverted Leica Sp8 TCS microscope using temperature and CO_2_ (5%) control, a 20x objective with NA = 0.7, and glycerol immersion. Excitation lasers were 488 nm and 561 nm, and signal was captured with HyD detectors. Twenty-five *z* planes were captured every 6 min; final image resolution was 0.4 × 0.4 × 0.57 μm. The tracking and analysis procedure is described in S1 Text section C2.

## Supporting information

S1 Fig Detailed description of the tree-structured ridge-following procedure. Illustration of the different phases of the tree-structured ridge-following procedure. Detailed description of the figure is available in S1 Text section A1. (A) Overview of the procedure for the construction of ridglet trees. In all diagrams, the signal of the steerable filter response is shown as grey lines. Ridglets are shown in red; currently selected maxima are represented as yellow circles, crossing points as blue circles, and leaf points as green circles. The ‘Pixel-level ridge following’ section represents the moving kernel in which a small square represents an image pixel. ‘Illegal’ pixels are shown with a red cross and ‘potential’ pixels with a question mark. (B) Illustrations of the stop conditions for the 2 phases of the ridglet creation process. For phase 2, a large field of view with 3 nuclei is drawn. Branches of the tree that have already been built are represented with red dashes; a red circle is drawn at the position of the root node. Green leaves are represented where leaf nodes are identified, and an orange leaf is drawn where a path intersection is found, as this induces a new leaf child in the corresponding internal node of the tree. C, own path crossing; CP, parent ridglet crossing point; D, distance stop; I, intensity drop; L, loop to self; M, missed; O, origin; P, path to parent node; T, sharp turns.
(EPS)

S2 Fig Shape creation and shape features. (A) Principle of 2D shape creation from a ridglet tree. The diagram on the left shows a ridglet tree for which the root is shown in blue, leaf ridglets are in green and are annotated with letters, and other ridglet nodes are coloured in yellow. The 6 shapes that can be created from the tree are drawn on the right-hand side. The 2-letter code on top of the shape indicates the leaf combination that the shape was constructed from. (B-C) Representations of the BC shape shown in (A) as it appears in a digital image in which each square in the grid illustrates a pixel. (B) The 2 categories of pixels used for the calculation of the shape’s intensity-based features. (C) The convention to code the outline of the shape as a chain used to calculate the shape’s curvature features (left panel). Calculated levels of curvature are shown in the centre panel, and the classification of pixels as either convex or concave is shown in the right panel.
(EPS)

S3 Fig Description of the depth linkage procedure and definition of user-defined variables. The schematics in this figure represent individually segmented planes in a 3D image. Image planes are shown as dashed vertical lines with 2D areas identified in the previous steps of the segmentation drawn as solid shapes. The relative position of each plane is indicated by the letter P followed by the plane index. (A) Example of a directional graph created by the first step of the procedure. Arrows represent the links that are created if the two connected shapes can potentially be part of the same volume. (B) Required conditions for link creation when the areas to look up (blue shapes) are located in the directly adjacent plane as the area under consideration (orange shape). The drawing at the top shows the max intercentroid distance threshold as a dashed circle. The red cross shows the centroids that are excluded, and green ticks show the centroid that can be included. The bottom drawing shows the effect of the ‘overlap threshold’. Percentages indicate how much of the area overlaps with the surface of the other area. Left percentage is for the blue shape, and right percentage is for the orange shape. The outcome with two distinct values for the overlap threshold is given. (C) Condition for edge creation when the area to look up (blue shape) is located farther than the plane directly adjacent to the area under consideration (orange shape). The ‘Max Jump’ variable defines the maximum number of planes that are allowed between two areas for an edge to be created. The drawing also shows the rule that applies when the area already possesses an edge with an area in a plane located upstream. (D) Diagrams showing rules applying when ‘ambiguities’ are detected. The shapes colours indicate their unique ID, and the area with ambiguities is shown in orange. Two cases are represented: (1) Ambiguity is found both with the above plane and with the plane below. In this example, the JI between the merged areas above and the current area is higher than the JI between the merged areas below and the current plane. (2) Ambiguity is found only in the plane below. The outcome obtained in the case that the JI between the current area and the merged areas in the bottom plane is higher than the JI between the current area and the area above is shown on the right. (E) Illustration of the volume-labelling procedure. Numbers in rounded squares indicate the order of events. The numbers next to the edges represent the value of the JI between two connected areas. Each colour indicates a unique ID. Areas with the same ID will belong to the same volume eventually. White means that no volume ID has yet been assigned. The effect of the ‘volume constraint’ (maximum volume and minimum volume variables) is shown. (F) Illustrations of the optional postprocessing steps. Left panel: Intensity-based splitting. Example image planes with LaminB1 signal are shown. The corresponding diagram illustrates a cut where the peak in the IR (intensity at the centre divided by intensity at the rim) is found. Middle panel: Displacement-based splitting—the top diagram shows an example of an undersegmented volume. The profile of the CD along image depth is given. In the bottom drawing, a cut is shown at the peak of the CD value. Right panel: Volume smoothing. Examples of artifacts are given in the top diagram. The bottom drawing shows how these artifacts are expected to be corrected. Additional areas at the tips of the volume are also shown. CD, centroid displacement; IR, intensity ratio; JI, Jaccard Index.
(EPS)

S4 Fig Overview of Nessys features for segmentation validation and benchmarking. (A) Snapshot of the first window to appear when launching the Nessys stand-alone application. This window lets the user choose the specific tool to work with. (B) Snapshot of the segmentation editor interface. The streak region of the E8.75 embryo (blue and white image) is loaded into the editor together with a segmentation result, which appears as yellow outlines. Selected shapes have been highlighted in red. (C) Overview of the ‘segmentation comparator’ tool. Boxes with a title over an orange background represent Java implementations in the application. The tasks that they handle are indicated inside the box. The ‘SegmentationComparator’ interface is shown in a blue box and can be extended to add custom metrics to the benchmarking process (dashed box). Required inputs are shown on the left-hand side: A GT image and a tested segmentation are loaded into the application, which performs ‘shape matching’ and error counting and computes performance metrics. Note that batch processing of images is supported as long as the number and dimensions of GT images are the same as tested segmentation images. Outputs of the program are illustrated on the right-hand side of the diagram. Summary.tsv is a table with all computed metrics for each image. The raw image of the 3D error map created by the program is shown with the ‘16 colours’ lookup table of ImageJ (Image Map, left) and a 3D view of this image obtained with the ImageJ 3D viewer (Image Map, right). Blue: accurate hit, yellow: spurious, purple: merge, orange: split. An online video tutorial for the Nessys method is readily available at https://pickcellslab.frama.io/docs/use/features/segmentation/nessys/, and detailed tutorials for all Nessys tools will be released soon on the following website: https://pickcellslab.frama.io/docs/. E, embryonic day; GT, ground truth; Nessys, Nuclear Envelope Segmentation System.
(EPS)

S5 Fig Histograms of the deviation of morphological features from the ground truth for each biological dataset including all nuclei. This figure provides data complementary to Fig 4C. Blue: Nessys, purple: Ilastik, yellow: MINS, green: Farsight. Data tables listing individual measurements used for the figure are available on GitLab (https://framagit.org/pickcellslab/data/2019_nessys). Nessys, Nuclear Envelope Segmentation System.
(EPS)

S6 Fig Nessys is robust to variations in fluorescence intensity and low signal-to-noise ratios. (A) Variation in intensity and Nessys results in the E8.75 zone 1 image (*z* slice 44). (B) Magnified views of the E8.75 zone 1 with increasing amount of Gaussian noise. (C-D) Nessys accuracy with increasing levels of noise expressed as Precision, Recall, and F-measure (C) or in terms of topological errors (D). The same variables were used throughout except where ‘parameters adjusted’ is shown. The data tables listing individual measurements used for the figure are available on GitLab (https://framagit.org/pickcellslab/data/2019_nessys). E, embryonic day; Nessys, Nuclear Envelope Segmentation System.
(EPS)

S7 Fig Higher sampling rates in the *z* axis help Nessys achieve high accuracy. Nessys accuracy with increasing *z*-step sizes expressed in terms of Precision, Recall, and F-measure (A) or in terms of topological errors (B). The *z* step size is indicated in μm, with the corresponding average number of *z* slices per nucleus shown underneath. The data tables listing individual measurements used for the figure are available on GitLab (https://framagit.org/pickcellslab/data/2019_nessys). Nessys, Nuclear Envelope Segmentation System.
(EPS)

S8 Fig Lower bit depth does not impact Nessys accuracy. Nessys accuracy for the E7.5 zone 1 image or E8.75 zone 1 image in 12- or 8-bit versions expressed in terms of Precision, Recall, and F-measure (A) or in terms of topological errors (B). The data table listing individual measurements used for the figure is available on GitLab (https://framagit.org/pickcellslab/data/2019_nessys). E, embryonic day; Nessys, Nuclear Envelope Segmentation System.
(EPS)

S9 Fig Nessys can segment non-NE signals with reasonable accuracy. (A) Representative images (top) and Nessys results (bottom) for non-NE signals. The source of the dataset/image is indicated between brackets. All images have been reproduced here with explicit permission from the authors. (B-C) Nessys accuracy for non-NE signals expressed in terms of Precision, Recall, and F-measure (A) or in terms of topological errors (B). The data table listing individual measurements used for the figure is available on GitLab (https://framagit.org/pickcellslab/data/2019_nessys). BBBC, Broad Bioimage Benchmark Collection; NE, nuclear envelope; Nessys, Nuclear Envelope Segmentation System.
(EPS)

S1 Movie Examples of Nessys segmentation outputs. Plane-by-plane animation of the Nessys segmentation outputs shown in Fig 1B and 1C. The LaminB1 signal is overlaid in cyan. Nessys, Nuclear Envelope Segmentation System.
(AVI)

S2 Movie Illustration of the ridge-following procedure. This is a slow-motion movie of the ridge-following procedure. The movie starts by a display of the final tree as described in the legend of Fig 2. The green portion of the tree represents the pair of leaves selected as the most probable valid shape according to the ranking performed by the classifier. Then, a sequence shows how the tree was grown. The green dot represents the location of the ridge-following procedure at a given time, and the blue lines highlight the ridges that have already been identified. Finally the leaves, the root, and the ‘winning’ ridge are highlighted before the segmented area is drawn.
(AVI)

S3 Movie Time lapse of double colour reporter cells during neural differentiation. Movie of the time-lapse experiment shown in Fig 7. Green: NE-mKate2 signal, red: Sox1-GFP; 1 sec = 38 min in real time. GFP, green fluorescent protein; NE, nuclear envelope; Sox1, SRY-box transcription factor 1.
(AVI)

S1 Table Summary of DISCEPTS image properties. This table summarises the image properties of the DISCEPTS dataset.
(PDF)

S2 Table Nessys variables used to segment the DISCEPTS dataset. This table contains the variables that were used to segment the DISCEPTS dataset. Nessys, Nuclear Envelope Segmentation System.
(PDF)

S3 Table Description of the measures provided by the segmentation benchmarking tool. This table describes the measures provided by the segmentation benchmarking tool.
(PDF)

S4 Table Segmentation accuracy measures for each biological specimen and method. This table reports segmentation accuracy measures for each biological specimen and method.
(PDF)

S5 Table Processing time for each image and method. This table reports processing times for each image and method.
(PDF)

S6 Table Nessys processing-time details with increasing image plane size or plane number. This table reports Nessys processing-time details with increasing image plane size or plane number. Nessys, Nuclear Envelope Segmentation System.
(PDF)

S7 Table Primary antibodies used in this study. This table lists the primary antibodies used in this study.
(PDF)

S1 Data Zip archive containing the training sets used to segment the DISCEPTS dataset with Nessys. Each .tsv file can be loaded into the Nessys interface to reuse a classifier. Nessys, Nuclear Envelope Segmentation System.
(ZIP)

S1 Text This document contains detailed information about the methods used in this study. (PDF)

## Abbreviations

AFU: arbitrary fluorescence units
AP: anterior–posterior
DIC: differential interference contrast
E: embryonic day
EMD: Emerin
ER: endoplasmic reticulum
ES: embryonic stem
FACS: fluorescence-activated cell sorting
FP: fluorescent protein
GFP: green fluorescent protein
GT: ground truth
ICM: inner cell mass
JI: Jaccard Index
Mpx: megapixels
NE: nuclear envelope
Nessys: Nuclear Envelope Segmentation System
NLS: nuclear localisation signal
Sox1: SRY-box transcription factor 1
Tcf15: transcription factor 15
TE: trophectoderm
