# Supplementary material for: Nessys: A new set of tools for the automated detection of nuclei within intact tissues and dense 3D cultures
Source: PLoS Biol. 2019 Aug 9;17(8):e3000388. doi: 10.1371/journal.pbio.3000388 (PMC6703695; doi:10.1371/journal.pbio.3000388)
Supplement: S1 Table — This table summarises the image properties of the DISCEPTS dataset. (PDF) [file pbio.3000388.s013.pdf]

S1 Table: Summary of DISCEPTS image properties

Biological Specimen	Number of images	Bit Depth	Channels	Imaging medium	Microscope	Objective	Voxel size (µm)
Neural Monolayer	1	8	GammaTubulin / LaminB1 / Sox1-GFP / Dapi	Prolong Gold	Leica SpE	ACS APO 63.0x1.30 OIL	0,227 x 0,227 x 0,503
3D Acini	3	12	Oct6 / LaminB1 / Dapi / gammaTubulin	Prolong Gold	Leica Sp8	HC PL APO 40x/1.30 Oil CS2	0.238 x 0.238 x 0.5
Blastocysts	5	12	LaminB1 / Dapi	PBS	Leica Sp8	HC PL APO 40x/1.30 Oil CS2	0.36 x 0.36 x 0.5
E7.5	1	12	LaminB1 / Dapi	BABB	Leica Sp8	HC PL APO 40x/1.30 Oil CS2	0.142 x 0.142 x 0.5
E8.75	1	12	Dapi / Tcf15-venus / gammaTubulin / LaminB1	BABB	Leica Sp8	HC PL APO 40x/1.30 Oil CS2	0.28 x 0.28 x 1
