# Supplementary material for: Nessys: A new set of tools for the automated detection of nuclei within intact tissues and dense 3D cultures
Source: PLoS Biol. 2019 Aug 9;17(8):e3000388. doi: 10.1371/journal.pbio.3000388 (PMC6703695; doi:10.1371/journal.pbio.3000388)
Supplement: S2 Table — This table contains the variables that were used to segment the DISCEPTS dataset. Nessys, Nuclear Envelope Segmentation System. (PDF) [file pbio.3000388.s014.pdf]

S2 Table: Nessys parameters used to segment the DISCEPTS dataset.

Step	Parameter	Neural Monolayer	Acini (WT and KO)	Acini (Het)	Blastocyst	E7.5	E8.75
**Denoising**	Method	None	None	None	None	None	None
**Steerable Filter**	staining type	enveloppe	enveloppe	enveloppe	enveloppe	enveloppe	enveloppe
	scale	2.75	2	2	2	1.2	1.5
	quality	Normal	High	High	Normal	Normal	Normal
**Maxima**	threshold	0.06	1	1.2	0.4	1.4	0.35
**Tracing**	search radius	4	3	3	3	3	3
	delta	0.14	0.1	0.3	0.1	0.15	0.1
	min radius	8	8	8	10	5	4
	max radius	40	45	40	30	25	20
**Shape ranking**	classifier	Neural_Classifier	Acini_Classifier1	Acini_Classifier2	Blastocysts_Classifier	E75_Classifier	E90_Classifier
**3D Linkage**	min volume	2500	6000	6000	6000	2000	1000
	max volume	35000	20000	25000	55000	8000	3500
	search radius	30	10	10	25	30	20
	min overlap	0.6	0.7	0.7	0.7	0.7	0.7
	allowed slice jumps	2	2	2	3	3	2
**Post-processing**	delete flat structures	yes	yes	yes	yes	yes	yes
	finalise	yes	yes	yes	yes	yes	yes
	split type	None	Directional (tolerance : 3)	Directional (tolerance : 3)	Intensity only (tolerance : 3)	None	Intensity only (tolerance : 3)
