# Supplementary material for: Nessys: A new set of tools for the automated detection of nuclei within intact tissues and dense 3D cultures
Source: PLoS Biol. 2019 Aug 9;17(8):e3000388. doi: 10.1371/journal.pbio.3000388 (PMC6703695; doi:10.1371/journal.pbio.3000388)
Supplement: S3 Table — This table describes the measures provided by the segmentation benchmarking tool. (PDF) [file pbio.3000388.s015.pdf]

S3 Table: Description of the measures provided by the segmentation benchmarking tool

**ClusteringComparator**	Name of the java class generating the measures listed below
Jaccard Index	Similarity measure (higher values are better)
Rand Index	Similarity measure (higher values are better)
Hausdorff Mean	maximum of the set of minimal distances between two compared shapes (lower values are better)
Hausdorff Std	Standard deviation of the Hausdorff measure distribution
NSD Mean	Normalised Sum of distances : average distance of mis-labelled pixels by the automated method from the border of the reference shape in GT (lower values are better)
NSD Std	Standard deviation of the NSD measure distribution
**SegmentationAccuracy**	Name of the java class generating the measures listed below
Merge count	Number of merged events
Merge size Mean	Average number of shapes per merge event
Merge size Std	Stand deviation of the distribution of the number of shapes per merge events
Miss	Number of miss events
Ref Number of Border Cells	Number of shapes touching the image border in the GT image
Ref Total Number of Cells	Total number of shapes in the GT image
Split count	Number of split events
Split size Mean	Average number of shapes per split event
Split size Std	Standard deviation of the distribution of the number of shapes per split events
Spurious	Number of spurious detection occurrences
Stretch	Number of detected shapes in the tested image which overlap with more than one GT shape (at least 25 % of detected shape volume). This can encompass merged or split events
Test Number of Border Cells	Number of shapes touching the image border in the tested image
Tested Total Number of Cells	Total number of shapes in the tested image
