# Supplementary material for: Nessys: A new set of tools for the automated detection of nuclei within intact tissues and dense 3D cultures
Source: PLoS Biol. 2019 Aug 9;17(8):e3000388. doi: 10.1371/journal.pbio.3000388 (PMC6703695; doi:10.1371/journal.pbio.3000388)
Supplement: S4 Table — This table reports segmentation accuracy measures for each biological specimen and method. (PDF) [file pbio.3000388.s016.pdf]

S4 Table: Segmentation accuracy measures for each biological specimen and method

Monoloayer (1 image - 470 Cells)

Method	JI	RI	Haussdorf	NSD	Merge	Miss	Split	Spurious	Accurate	Precision	Recall	F-Measure
Nessys	0,61	0,94	256,70	0,30	4,50 %	2,00 %	2,84 %	1,03 %	89,63 %	96,13 %	93,48 %	94,79 %
Ilastik	0,58	0,94	261,47	0,44	10,00 %	4,25 %	19,69 %	2,21 %	63,85 %	78,10 %	86,10 %	81,90 %
MINS	0,42	0,91	256,36	0,64	16,75 %	3,25 %	9,25 %	8,75 %	62,00 %	82,00 %	80,39 %	81,19 %
FarSight	0,63	0,93	256,11	0,18	24,25 %	0,00 %	21,32 %	0,76 %	53,67 %	77,92 %	75,99 %	76,94 %

Acini (3 images - 512 Cells)

Method	JI	RI	Haussdorf	NSD	Merge	Miss	Split	Spurious	Accurate	Precision	Recall	F-Measure
Nessys	0,68	0,97	91,55	0,14	5,76 %	0,00 %	3,66 %	1,29 %	89,30 %	95,05 %	94,24 %	94,65 %
Ilastik	0,53	0,96	92,21	0,33	27,30 %	4,26 %	23,45 %	3,13 %	41,86 %	71,75 %	68,85 %	70,27 %
MINS	0,41	0,95	92,11	0,52	28,94 %	2,35 %	19,75 %	3,57 %	45,39 %	75,06 %	69,95 %	72,41 %
FarSight	0,58	0,96	92,95	0,14	39,25 %	0,00 %	8,71 %	19,58 %	32,47 %	70,42 %	62,14 %	66,02 %

Blastocysts (5 images - 699 Cells)

Method	JI	RI	Haussdorf	NSD	Merge	Miss	Split	Spurious	Accurate	Precision	Recall	F-Measure
Nessys	0,69	0,96	80,37	0,17	0,17 %	4,67 %	2,57 %	0,00 %	92,59 %	97,43 %	95,15 %	96,28 %
Ilastik	0,73	0,97	80,80	0,16	1,17 %	1,34 %	4,75 %	0,65 %	92,10 %	94,60 %	97,48 %	96,02 %
MINS	0,71	0,96	81,34	0,21	3,51 %	0,17 %	4,68 %	1,78 %	89,87 %	93,40 %	96,33 %	94,84 %
FarSight	0,70	0,95	78,05	0,03	15,66 %	0,00 %	3,63 %	1,83 %	78,89 %	94,55 %	84,65 %	89,32 %

E7.5 (2 Zones 924 Cells)

Method	JI	RI	Haussdorf	NSD	Merge	Miss	Split	Spurious	Accurate	Precision	Recall	F-Measure
Nessys	0,59	0,87	283,01	0,17	6,27 %	2,34 %	4,16 %	0,35 %	86,88 %	95,49 %	91,44 %	93,42 %
Ilastik	0,70	0,90	272,92	0,09	6,34 %	0,00 %	6,86 %	0,66 %	86,14 %	92,48 %	93,64 %	93,05 %
MINS	0,54	0,87	283,50	0,47	14,71 %	1,67 %	22,69 %	2,87 %	58,07 %	74,44 %	84,10 %	78,98 %
FarSight	0,60	0,83	123,24	0,01	18,57 %	0,00 %	28,81 %	2,71 %	49,91 %	68,48 %	82,67 %	74,91 %

E8.75 (3 zones - 4593 Cells)

Method	JI	RI	Haussdorf	NSD	Merge	Miss	Split	Spurious	Accurate	Precision	Recall	F-Measure
Nessys	0,64	0,85	991,02	0,26	3,11 %	3,03 %	2,05 %	0,81 %	91,00 %	97,14 %	93,81 %	95,45 %
Ilastik	0,65	0,86	988,70	0,34	8,15 %	2,92 %	6,28 %	1,28 %	81,38 %	92,44 %	88,84 %	90,61 %
MINS	0,56	0,82	1004,26	0,46	13,10 %	0,55 %	16,29 %	2,89 %	67,17 %	80,82 %	86,55 %	83,59 %
FarSight	0,63	0,84	1033,03	0,18	21,30 %	0,30 %	7,11 %	2,51 %	68,79 %	90,39 %	78,43 %	83,98 %
