# Supplementary material for: Nessys: A new set of tools for the automated detection of nuclei within intact tissues and dense 3D cultures
Source: PLoS Biol. 2019 Aug 9;17(8):e3000388. doi: 10.1371/journal.pbio.3000388 (PMC6703695; doi:10.1371/journal.pbio.3000388)
Supplement: S5 Table — This table reports processing times for each image and method. (PDF) [file pbio.3000388.s017.pdf]

S5 Table: Processing Time for each image and method

	Image	Zone	Image Size (Mpx)	Image Planes	XY Res (µm)	Nessys	Ilastik	Farsight	Mins
**Monolayer**	130520_Rosette	Whole image	47,012364	36	0,23	101	232	59	702
**Acini**	E14tg2a_d2_6.tif	Whole image	40,894464	39	0,24	87	168	660	499
	Tcf15-KO-A1_d2_3.tif	Whole image	37,748736	36	0,24	75	148	521	432
	Tcf_Het_d2_5.tif	Whole image	28,311552	27	0,24	37	251	310	325
**Blastocysts**	161202_B1_C1	1	17,5879	236	0,36	59	116	34	NA
	161202_B1_C2	2	17,254904	236	0,36	67	123	33	157
	161202_B2_C1	1	31,349175	237	0,36	97	163	61	333
	161202_B2_C2	2	10,953192	237	0,36	61	79	21	151
	161202_B3	Whole image	21,826675	239	0,36	67	104	42	209
	161202_B4_C1	1	12,77804	257	0,36	75	76	25	168
	161202_B4_C2	2	20,509628	257	0,36	84	128	40	190
	161202_B4_C3	3	13,65441	257	0,36	69	74	27	118
	161202_B5_C1	1	12,27447	196	0,36	64	66	24	107
	161202_B5_C2	2	11,56077	196	0,36	63	62	23	98
	161202_B5_C3	3	11,56077	196	0,36	67	62	23	102
**E7.5**	161202_E75_Zone1	1	3,8794	140	0,2838	63	26	6	57
	161202_E75_Zone2	2	8,22987	297	0,2838	132	52	12	157
**E8.75**	E90_Epithelia	Epithelia	12,0048	41	0,28	38	79	171	345
	E90_Somite_64_124	Somites	7,9422	61	0,28	29	39	165	174
	E90_Streak	Streak	7,095222	51	0,28	40	42	115	158
