# Supplementary material for: Nessys: A new set of tools for the automated detection of nuclei within intact tissues and dense 3D cultures
Source: PLoS Biol. 2019 Aug 9;17(8):e3000388. doi: 10.1371/journal.pbio.3000388 (PMC6703695; doi:10.1371/journal.pbio.3000388)
Supplement: S6 Table — This table reports Nessys processing-time details with increasing image plane size or plane number. Nessys, Nuclear Envelope Segmentation System. (PDF) [file pbio.3000388.s018.pdf]

S6 Table: Nessys processing time details with increasing image plane size or plane number

		Image Size			Image Properties			Processing time (Sec.)					
	Image	X	Y	Z	Mpx	Cell Number	Cells/Mpx	Steer	RBS	Linkage	Total	Mvx/Sec	Cells/Sec
Increasing Plane Size	A1	472	359	145	24,56996	4511	183,6	3	105	21	129	0,190	35,0
	A2	665	505	145	48,694625	7956	163,4	6	172	34	212	0,230	37,5
	A3	940	715	145	97,4545	15280	156,8	12	276	63	351	0,278	43,5
	A4	1330	1011	145	194,97135	23542	120,7	24	428	104	556	0,351	42,3
	A5	1881	1430	145	390,02535	35443	90,9	48	649	165	862	0,452	41,1
	A6	2660	2022	145	779,8854	61540	78,9	102	1080	327	1509	0,517	40,8
	A7	3763	2860	145	1560,5161	81284	52,1	199	1550	502	2251	0,693	36,1
Increasing Plane Number	B1	1024	1024	24	25,165824	4032	160,2	6	81	21	108	0,233	37,3
	B2	1024	1024	48	50,331648	7892	156,8	10	158	42	210	0,240	37,6
	B3	1024	1024	96	100,6633	15485	153,8	21	302	72	395	0,255	39,2
	B4	1024	1024	192	201,32659	30676	152,4	41	602	144	787	0,256	39,0
	B5	1024	1024	384	402,65318	61108	151,8	81	1270	281	1632	0,247	37,4
	B6	1024	1024	580	608,17408	92250	151,7	113	1827	556	2496	0,244	37,0
