# Supplementary material for: Nessys: A new set of tools for the automated detection of nuclei within intact tissues and dense 3D cultures
Source: PLoS Biol. 2019 Aug 9;17(8):e3000388. doi: 10.1371/journal.pbio.3000388 (PMC6703695; doi:10.1371/journal.pbio.3000388)
Supplement: S7 Table — This table lists the primary antibodies used in this study. (PDF) [file pbio.3000388.s019.pdf]

S7 Table: Primary antibodies used in this study

Epitope	Host	Dilution	Suplier	Reference
Lamin B1	Rabbit	1/1000	Abcam	ab16048
Gamma-Tubuli	Mouse	1/250	Abcam	ab11316
GFP	Chicken	1/1000	Abcam	Ab 13970
Oct6	Goat	1/200	Santa Cruz	sc-11661
Nuclear Pore	Mouse	1/1000	Abcam	ab24609
