# Supplementary material for: Nessys: A new set of tools for the automated detection of nuclei within intact tissues and dense 3D cultures
Source: PLoS Biol. 2019 Aug 9;17(8):e3000388. doi: 10.1371/journal.pbio.3000388 (PMC6703695; doi:10.1371/journal.pbio.3000388)
Supplement: S1 Text — (PDF) [file pbio.3000388.s021.pdf]

S1 TEXT

A. Detailed description of the segmentation method

An overview of the segmentation procedure is depicted in Fig 2. This section provides a more detailed description of some of the key steps included in the segmentation method. Please also note that source code and the latest documentation (including advice on how to adjust parameters) can be found on the Nessys online repository:

https://framagit.org/pickcellslab/nessys

A.1. Tree-structured ridge following procedure (Fig S1)

The purpose of this step is to identify outlines of nuclei within a given 2D image plane.

The key idea is that ridges are built incrementally to form a series of 'ridglets' organised hierarchically in a tree until some stop conditions are met. One tree is built per maximum detected in the previous step. Such a 'ridglet tree' can then be used to build a list of 2D shapes. The 'best' shape is then selected and drawn in the result image (See section A.2.)

Fig S1 depicts the procedure which generates the tree and Movie S2 shows a real example of this task in slow motion.

Inputs received from the previous steps include:

- The original NE signal for a specific plane
- The corresponding ridge-enhanced plane (steerable filter output)
- Image locations to use for initialisation of the trees (maxima identified in the previous step)

Basic procedure:

The tree building process starts by the selection of a unique location in the 2D plane (Fig S1A-1). Maxima are initially sorted in descending order of signal intensity. Next, pixel intensities are collected along the perimeter of a circle centered on the selected maximum (Fig S1A-2).

Intensity peaks in this intensity profile are detected to generate new ridge points from which

ridges are traced back towards the center of the circle. This system makes it possible to “jump” over gaps in the ridge signal (see also Fig S1B). The radius of the ‘search circle’ is given by the parameter ‘**search radius**’ in the Nessys interface. A larger radius allows in principle a larger gap to be crossed.

At this point only half-ridglets are created. In a second phase, the ridges are traced outward (Fig S1A-3) until stop conditions are met (as explained further below). Once the outward tracing stops, a full ridglet is built. As mentioned earlier, ridglets are maintained in a tree structure so the origin can be traced back from any ridglet. Finally, the most outward points of the ridglets are turned into new search circle centres and the procedure is repeated until the full ridglet tree is constructed (Fig S1A-4).

Pixel-level ridge following:

At the pixel level, ridges are followed using a moving kernel system (Fig S1- top right). The kernel includes 4 pixel categories.

- The center pixel C which is the current location of the tracing kernel
- The ‘rear’ pixel R which defines the direction of the tracing process (R is located behind C).
- ‘Illegal’ pixels which are neighbour pixels which cannot become the next ridglet point. This prevents ridglets to form turns that are too sharp.
- ‘’potential’ pixels which define the next possible ridglet points.

The location of R and C are defined during initialisation and depends whether the following procedure occurs during phase 1 or phase 2 of the ridglet creation. During tracing, the kernel moves sequentially to the brightest of the 3 ‘potential’ pixels. Indeed, ridges are brighter towards their center and dimmer on the sides. With this system and knowledge of the location of the rear pixel, only 3 pixels are read at each iteration, making the procedure particularly efficient.

Fail and success conditions:

Stop conditions for the ridge following procedure during phase 1 and phase 2 of ridglet creation are shown in Fig S1B.

During phase 1, there are 4 possible outcomes:

- The tracing process finishes at origin (O in Fig S1B). In this case the half-ridglet is constructed with its parent ridge point maintained at the initial origin point.
- The tracing process stops because the ridge intensity drops below a given threshold (I in Fig S1B). In this case, the ridglet is discarded. Note: the threshold is defined by the '**delta**' parameter where threshold = delta x starting ridge point intensity.
- The trace moves outside the 'probing' circle and never reaches the origin (M in Fig S1B). In this case, the trace is corrected by connecting the trace to the origin at its closest point in the path.
- The trace reaches the parent ridglet before reaching the origin (P in Fig S1B). In this case the ridglet is maintained and the origin for all successful ridglets is rebased where the trace reaches the parent ridglet. If multiple traces of this type occur, the rebase point is chosen to be the closest from the parent ridglet crossing point.

Note that when this step if performed without a parent (first iteration), only I and O are allowed.

During phase 2, four outcomes are again possible:

- The trace stops because it has reached a maximum distance from its starting point (D in Fig S1B). In this case, the full ridglet is constructed, it is maintained in the tree and its last point will be used as a new origin for the next iteration. (The maximum distance is defined by the search radius parameter)
- The tracing process stops because the ridge intensity drops below a given threshold (as in phase 1, the threshold is defined by the delta parameter). Here the full ridglet is completed and maintained but it is set as a leaf in the tree and will not be used in the next iteration.
- The trace forms a loop and reaches its other half-ridglet (L in Fig S1B). In this case the ridglet is maintained but the trace from phase 2 is discarded. The half-ridglet from phase 1 is set as a leaf in the ridglet tree.
- The trace reaches an ancestor ridglet (not its direct parent – C in Fig S1B). In this case the ridglet is constructed, maintained in the tree and set as a leaf. In addition, the point

reached in the ancestor ridglet is also set as an extra leaf in the tree. This is to make it possible to form a 2D shape using the trace connecting these 2 points in the next step (see section B2.)

Finally, a ridglet will also be tagged as 'leaf' if the maximal tree depth has been reached. This maximal depth is function of the "max radius" parameter. This stop condition is useful to avoid the propagation of the tracing procedure too far away from its origin.

A.2. 2D Shape creation and selection with a Naive Bayes Classifier

<u>Shape creation:</u>

The previous step, generates a ridglet tree. From this tree, it becomes possible to construct a list of 2D shapes using all leaf pair combinations. An example is shown in Fig S2A where each shape is created by connecting the two leaf ridge points together with a straight line and by following the ridglets up to their least common ancestor in the tree.

<u>Shape pre-selection:</u>

At this point a 'minimal validity test' is performed for each shape using simple geometric features. This makes it possible to pre-filter the shapes before computing more complex features and thus to speed up the process of shape selection.

A shape T passes the test if it conforms to the following constraints:

1. $P_{min} < P_{shape} < P_{max}$

   where P_{min} is the perimeter of a circle with user-defined min radius, P_{shape} is the perimeter of the tested shape and P_{max} is the perimeter of a circle with user-defined max radius.
2. P_{shape} must be 8 times greater than the distance between the two leaf ridge points.
3. The compactness of the shape must be > 4.
4. If the tested shape T overlaps with another existing shape E, then the area of the intersection must be less than 20% of the area of T and less than 20% of the area of E.
5. Finally if overwriting the intersecting portion of E with T leads to E becoming smaller than the minimal size specified by 'min radius', then T fails the test.

Note: this check contains a few constants that were adjusted during the development of the software based on preliminary results with test samples (acini images). We appreciate that this may be sub-optimal and may constitute a point for improvements in the future.

Shape features for classification and ranking:

After the pre-selection step described above, additional features are computed for the remaining shapes:

These features are described in the table below.

IQR: Inter-Quartile Range, k: curvature computed from the chain code as described in [1]. For a definition of Ridge, inner area, concave or convex points in the shape, please see Fig S2 B and C.

	Description	Name in the training set	Definition	Notes
Intensity-Based Features	Normalised Median to Mean Difference of the intensities on the enhanced image along the ridge	Border DMean Steered	$\frac{\lvert(Median_{(Ridge)} - Mean_{(Ridge)})\rvert}{Mean_{(Ridge)}}$	Measures how skewed the distribution of itensities are along the ridge in the filter response image
	Normalised Interquartile Range of the intensities distribution on the enhanced image along the ridge	Border IQR Steered	$\frac{IQR_{(Ridge)}}{Mean_{(Ridge)}}$	Measures how variable the signal of the filter response is amongst ridge pixels
	Normalised Median to Mean Distance of the intensities on the input image along the ridge	Border DMean Channel	$\frac{\lvert(Median_{(Ridge)} - Mean_{(Ridge)})\rvert}{Mean_{(Ridge)}}$	Measures how skewed the distribution of itensities are along the ridge in the original NE signal
	Normalised Interquartile Range of the intensities distribution on the input image along the ridge	Border IQR Channel	$\frac{IQR_{(Ridge)}}{Mean_{(Ridge)}}$	Measures how variable the signal of the original NE signal is amongst ridge pixels
	Normalised Median to Mean Distance of the intensities on the input image inside the polygon defined by the ridge	Inner DMean Channel	$\frac{\lvert(Median_{(Inner\ Area)} - Mean_{(Inner\ Area)})\rvert}{Mean_{(Inner\ Area)}}$	Measures how skewed the distribution of itensities are within the inner area of the shape in the original NE signal
	Normalised Interquartile Range of the intensities distribution on the input image inside the polygon defined by the ridge	Inner IQR Channel	$\frac{IQR_{(Inner\ Area)}}{Mean_{(Inner\ Area)}}$	Measures how variable the signal of the original NE signal within the inner area of the shape
Curvature Features	Compactness	Compactness	$\frac{Area - Perimeter}{Area - \sqrt{Area}}$	Measures how similar the shape is to a circle. Values close to one indicate an quasi-circular shape.
	Proportion of ridge pixels where the local curvature is zero	Curvature Zeros	$\frac{Number\ of\ Ridge\ points\ with\ k=0}{Total\ Number\ of\ Ridge\ points}$	Measures how much of the shape outline is composed of straight lines
	Average of the absolute curvature over the non zero curvature pixel	Curvature non Zero	$\frac{\sum k_{Convex} - \sum k_{Concave}}{N(Ridge\ with\ k=0)}$	Measures how 'intense' is the convexity of the shape
	Sum of curvature values over the concave points along the ridge	Sum Concave	$\sum k_{Concave}$	Measures the level of concavity in the contour of the shape
	Sum of curvature values over the convex points along the ridge	Sum Convex	$\sum k_{Convex}$	Measures the level of convexity in the contour of the shape

Final decision on shape selection:

The features described above are used for classification and ranking of the shapes.

For this purpose, we built a Gaussian Bayes classifier using the 11 shape features described in

the previous section. We chose to use a Gaussian Bayes classifier because of its simplicity and because Gaussian Bayes classifiers are known to be well suited to classify large datasets with minimal amount of training data. (training data used in this study are available as .tsv files in supplementary information).

1. Training:

For training, the classifier is initialised with equiprobable priors for two classes ‘valid’ and ‘invalid’ equal to 0.5. The training dataset can be generated interactively in the graphical user interface of Nessys (see the online video tutorial) or by loading an existing training set (we provide training sets used in this study in supplementary information).

In the interface we have set to 10 the minimum number of shapes of each class for considering the classifier to be valid.

In practice, we obtain satisfactory results with a training set consisting of 90 shapes on average. Selecting shapes in the Nessys interface is a rapid process, this corresponds roughly to 2 to 5 min in terms of time spent in the Nessys interface

2. Ranking

During segmentation, the shapes that remain after the ‘minimal validity check’ are associated with a score. This score is defined as the posterior probability of the shape being valid (pValid) (using the 11 feature vector) or as the negative of pInvalid (posterior probability of the shape being invalid) if pInvalid > pValid.

Once the score has been computed for all shapes of a tree, the shape with the highest score is selected. It is drawn in the result image only if its score is positive.

A.3. Depth linkage procedure

Once all planes have been segmented, individual areas are linked together into 3D volumes (Fig S3). The procedure is as follows:

- We first create a directed graph (V, E) of potential connections where V are areas and E define potential linkage (Fig S3 A). The required conditions are:
    - Create an edge E to the shape in the next plane if:

        - the inter-centroid distance is less than threshold (Fig S3 B - top)
        - the shape overlap is more than the minimum percentage of each area (Fig S3 B - bottom)
    - Create an edge E to the shape in the subsequent planes if
        - the number of 'jumped' planes is less than a given threshold (Fig S3 C - left)
        - the shape is not already linked to other areas in previous planes (Fig S3 C - right)

  When an edge is created, 'matching' features are computed and assigned to the edge. So they can be used for edge ranking during the graph colouring step (see further below). These features include:

        - the inter-centroid distance
        - the jaccard index
        - the sum of percentage of overlap of the two shapes
        - the barycentre of the intersection of the two shapes

- Once the graph is initialised, we look for regions of ambiguity within the graph (vertices with more than 1 incoming or outgoing edge, (Fig S3D)). These 'ambiguous cases are resolved according to the following rules:
    - if multiple connections are found both above and below the area, then a correction is performed which leads to the splitting of the area (Fig S3D). To decide how the area is split, the JI is computed between the area and the union of adjacent shapes in the above plane or in the plane below the area. The comparison of JI defines which of the above or below shapes will be copied in place of the current area (Fig S3D). NB: to account for this correction in subsequent steps, the newly created edges are assigned the 'winning' JI.
    - If multiple connections are found above the area only, the JI is computed between the area and the union of adjacent shapes in the above planes. If this new JI is higher than the JI found in the connection below, then the area is split (replaced by a copy of the shapes found in the above plane). Otherwise, no correction is applied.
    - If multiple connections are found below the area only, then the procedure described in the previous point is applied, only in the opposite orientation.
- After ambiguities have been resolved, areas are assigned a 'volume colour' using a graph colouring procedure (Fig S3E). This step is described below:

- Edges are sorted in descending value of the JI. (Most similar areas are colored first).
- Then, for each edge, we check the colour of the source and target area:
  - Case 1: None have a colour: we initialise a new volume by creating a new unique colour and assigning this colour to both areas.
  - Case 2: Only 1 has a colour: we test if the volume resulting from adding the non coloured area would be within user-defined bounds. If it is, the non-coloured area is assigned the same colour as the known volume. Otherwise, The non-coloured area is given a new unique colour and a new volume is initialised.
- Finally, small volumes (smaller than user-defined bounds) that remain in the graph at the end of the procedure are merged to larger adjacent volumes if the resulting volume remains inferior than the average volume found in the graph +/- 3 sigmas.

- At the end of the procedure, optional post-processing steps are available to refine the resulting segmentation (Fig S3F):
  - Intensity-based split: This procedure attempts to identify merged nuclei based on the profile of the signal intensity along the z axis (Fig S3F - left). When two nuclei are merged together in the z axis, intensity is expected to peak close to the centre of the volume. The volume is split if the two resulting volumes are within user-defined bounds.
  - Displacement-based split: This step attempts to find merged nuclei based on the rate of displacement of the centroids of the areas forming the volume when iterating along the z axis (Fig S3F – middle). When two nuclei are merged, the rate of displacement is expected to peak where the volume needs to be split. Again, the volume is split if the two resulting volumes are within user-defined bounds.
  - Volume smoothing: This step uses 3D morphological filters (closing operation followed by an opening operation) in order to fill holes and to smooth the resulting 3D shape (Fig S3F – right). Finally, tips are added at the bottom and top of the shape using a dilation in the z axis with an offset to place the tip along the main axis of the shape.

NB: This procedure is subject to change as we may further improve the method over time. We have setup an issue tracker (https://framagit.org/pickcellslab/nessys/issues) in our GitLab

repository which will enable anyone to look at scheduled plans to improve Nessys and also to propose changes and fixes.

B. Complementary details for the performance assessment procedure

B.1. Error counting

The steps outlined below describe the procedure implemented in the segmentation comparator module to identify segmentation errors and accurate detections:

1. For each shape in the GT image, we associate the shape in the tested segmentation image which possesses the biggest overlap. A reference of each matching pair is kept in memory as we iterate over GT shapes(forward match).
2. The same is done in the opposite direction: for each shape in the tested image, we identify the best matching shape in the GT image. The reference for each matching pair is kept in memory (backward match)
3. Shapes are then classified as follows:
    a. Miss: the GT shape shares less than 5% of overlap with its forward match (95% of the shape is background in the test image)
    b. Spurious: the tested shape shares less than 5% of overlap with its backward match (95% of the shape is background in the GT image)
    c. Merge: There is more than one forward match for the given GT shape in the tested image
    d. Split: There is more than one backward match for a given tested shape in the GT image.
    e. Accurate: There is exactly one backward match and one forward match for a pair of GT/tested shape.

NB: Shapes are excluded from the analysis if the matching shape in the GT image is in contact with any of the 6 image borders.

For each GT/Tested image pair given as input to the program, an image with the same

dimensionality of the input image is generated. This output image represents a map of the shape classes defined above. GT and detected nuclei are drawn as an outline and given a label which indicates their class as follows: ACCURATE = 1, EXCLUDED = 2, MISS = 3, SPURIOUS = 4, SPLIT = 5, MERGED = 6. Applying a look up table to this image such as the '16-colours' Lut in imageJ allows to visualise the accuracy of the segmentation method (Fig S4 C, see also Fig 3)

B.2. Precision and recall

Precision and recall were computed in LibreOffice calc. based on the output of the segmentation comparator.

Precision is defined as $\frac{TP}{TP+FP}$ and Recall as $\frac{TP}{TP+FN}$

TP (true positives) is defined as TP = $Ntest-FP$

where *Ntest* is the total number of shapes in the test image minus the number of shapes excluded from the analysis and FP (false positives) $FP=Nspur+Nsplit\times(\overline{Nfrag}-1)$

where *Nspur* is the number of spurious shapes, *Nsplit* is the number of split event and Nfrag is the average number of fragments per split shape.

FN (false negatives) is defined as $FN=Nmiss+Nmerge\times(\overline{Nms}-1)$

where *Nmiss* is the number of miss event, *Nmerge*, the number of undersegmentation event and *Nms* the average number of shapes per merge event.

B.3. Morphometric analysis

We performed the computation of morphometric features in a software built in our lab called PickCells (Blin et al., in preparation, source code is available at https://framagit.org/pickcellslab) and exported features to R [2] in order to build the plots shown in Fig 4C and Fig S5. Features were computed as follows:

- **Anisotropy:**

To measure the anisotropy for a given 3D shape, we computed the covariance matrix from the list of voxel coordinates of the shape and performed its eigen-decomposition. Our code uses the Math3 Apache library (http://commons.apache.org/proper/commons-math/). Anisotropy was defined as: $A = 1 - \frac{\lambda 2 + \lambda 3}{2\lambda 1}$ where lambda 1, 2 and 3 are the 1st, 2nd and 3rd eigenvalues of the decomposition respectively. The 'anisotropy difference' shown in Fig 4C is the anisotropy value of the tested shape minus the anisotropy value of the matching GT shape.

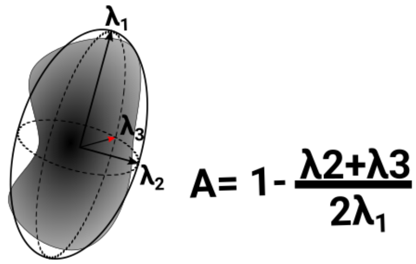

- **Eigen vector angles**

We computed 3D eigen vectors from the eigen-decomposition of the covariance matrix of the list of 3D coordinates. The eigen vector angle shown in Fig 4C is the angle between the first eigen vector of the tested shape and the first eigenvector of the GT shape expressed between 0 and 90°.

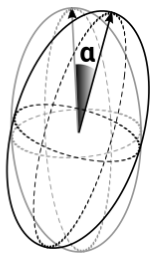

- **Jaccard Index**

The Jaccard Index (JI) between a tested shape and its matching GT shape was computed by expressing volumes (total volume and volume of the shapes intersection) as a number of

voxels. JI was defined as $JI = \frac{I_{GT}}{V_G + V_T - I_{GT}}$ where I_{GT} is the volume of the intersection between the GT and the tested shape, V_G is the volume of the GT shape and V_T is the volume of the tested shape.

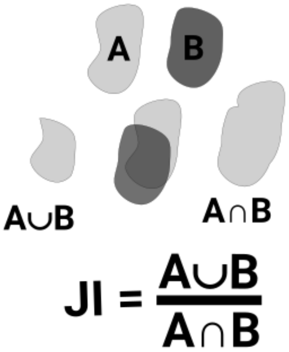

- **Intensity distance**

To simulate a heterogeneous expression of 3 transcription factors, we used the 32bit manually segmented output and applied the following procedure in ImageJ:

- Apply Lut 'Random_RGCor'
- Convert to RGB
- Gaussian blur (std = 1/3 of cell radius)
- Unsharp Mask (sigma = 6 and strength = 0.6)
- Add Gaussian noise with a standard deviation equals to 10.

We then converted the image to RGB to obtain 3 channels. To compute the intensity distance, we then measured the average intensity for each channel to obtain a 3D 'colour coordinate' for each shape. The 'intensity distance' shown in Fig 4C is the euclidian distance between the 3D colour coordinates of the tested shape and its best matching GT shape.

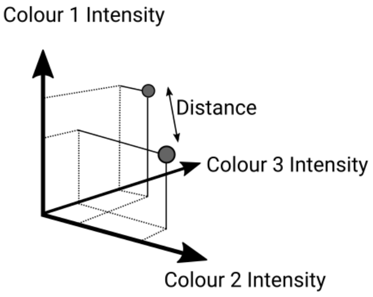

- **Inter-centroid distance:**

The inter-centroid distance corresponds to the euclidian distance between the centroid of the GT shape and the best matching tested shape.

- **Relative volume difference**

The relative volume difference was defined as $V_{dif} = \frac{V_T - V_G}{V_G}$

- **Neighbours difference**

To identify neighbours for each individual shape in an image, we first created a Delaunay triangulation using the centroids of the shapes as input. We then removed edges in the graph by applying a cutoff of 5µm to the shortest border to border distance between adjacent shapes. We validated the method visually by representing the data in a 3D scene in PickCells (Blin et al. In preparation) as shown in the image below where neighborhood is indicated as yellow arrows

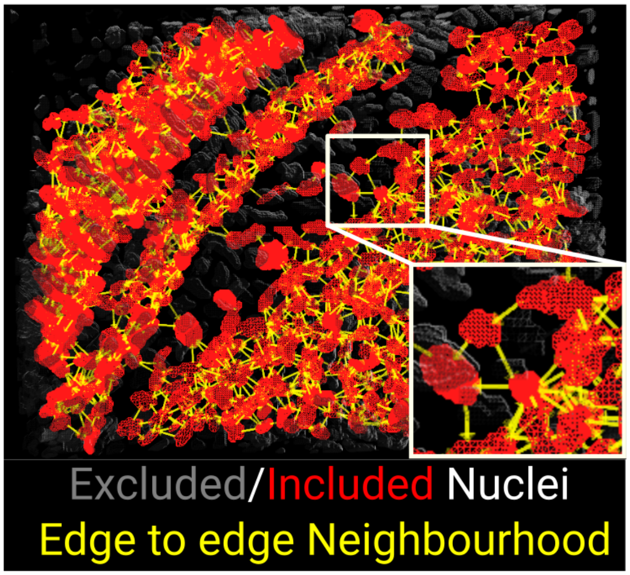

The number of neighbours for a given shape was given by the degree at the corresponding vertex in the resulting graph. The neighbours difference reported in Fig 4C and Fig S5 was equal to the number of neighbour of the tested shape minus the number of neighbour of the GT shape.

C. Details of the procedure to vary image quality

C.1. Noise

To generate a series of images with increasing level of noise, we used a cropped region in the bottom left corner of the E8.75 zone 1 benchmark image. Cropping was necessary to isolate nuclei with sensibly similar levels of intensities and obtain a signal to noise ratio relatively homogeneous across the image. This region contained 238 nuclei (excluding border nuclei) and the signal for the dimmest cells was roughly equal to 1000 AFU (12-bits image).

To generate noise in the image, we used the 'Add specified noise' command in ImageJ and chose Gaussian noise with varying standard deviations ranging from 50 to 500. This number is reported in figure S6 B. Parameters for segmentation were the same as shown in table S2 for E8.75, except for the 'adjusted parameter' data point (Fig S6 C and D). In this later case, the denoising step was applied (3D Gaussian filter with radius 0.5 x 0.5 x 1) and the classifier was updated (10 new invalid shapes and 19 valid shapes - Noise_Classifier in sup material)

C.2. Z-step size

To prepare images with varying z-step sizes, we used the E8.75 zone 1 image and resampled the image and the corresponding manual segmentation in ImageJ:

We used the 'scale' command with 'bilinear' interpolation for the color image and no interpolation for the segmentation. In both cases, we ensured that the box 'average when downsizing' was unticked.

For re-segmentation of these images, we used the parameters indicated in Table S2 for E8.75 except for the changes indicated below. Note that the min and max volume in the 3D linkage step needed to be adjusted to account for the loss of volume (in pixels) with increasing z-step size.

	Step Size (µm)						
**Parameter**	**0.5**	**0.71**	**1**	**1.275**	**1.7**	**2.55**	**5.1**
min volume	2000	1500	unchanged	500	400	300	150
max volume	7000	4000	unchanged	2200	2000	1600	1100
delete flat structures	unchanged	unchanged	unchanged	unchanged	unchanged	unchanged	no
finalise	unchanged	unchanged	unchanged	unchanged	unchanged	unchanged	no
split type	unchanged	unchanged	unchanged	unchanged	unchanged	None	None

C.3. Bit Depth

We modified the bit depth of the E7.5 zone 1 and E8.75 zone 1 images using ImageJ. The parameters for segmentation were maintained the same as for the 12bits original versions except for the maxima threshold which needed to be lowered in the 8 bits case. (0.4 instead of 1.4 for E7.5 and 0.05 instead of 0.35 for E8.75).

D. Segmentation of Dapi, DIC and membrane labelled samples

We used the complete image set BBBC039v1, Caicedo et al. 2018, for 2D Dapi signal and the first 10 images of BBBC030v1, Koos et al. 2016, for DIC signal available from the Broad Bioimage Benchmark Collection (BBBC) [3]. For 3D nuclei content and membrane signals we used the image distributed as part of the RACE application [4]. All images were used with explicit consent from the authors.

For BBBC030v1 we generated GT segmentations using the editor supplied with Nessys (as opposed to outlines which are provided with the dataset. We manually segmented the first 10 images and Nessys performance was assessed on these 10 images only). We also generated GT for the RACE image.

For segmentation with Nessys, we used the parameters listed in the table below

Step	Parameter	BBBC030v1	BBBC039v1	3D Nuclei	3D Memb.
**Denoising**	Method	Gaussian (radius : 2 x 2)	None	Gaussian (radius : 1 x 1 x 1)	Gaussian (radius : 1 x 1 x 1)
**Steerable Filter**	staining type	enveloppe	nuclear content	nuclear content	nuclear content
	scale	5	0.5	0.5	1
	quality	High	High	Highest	Normal
**Maxima**	threshold	0.02	4	1	0.5
**Tracing**	search radius	5	4	3	5
	delta	0.5	0.3	0.05	0.01
	min radius	0.2	10	4	4
	max radius	19	2500	12	12
**Shape ranking**	classifier	40	BBBC039v1_Classifier	3D_Dapi_Classifier	3D_Memb_Classifier
**3D Linkage**	min volume	NA	NA	400	1000
	max volume	NA	NA	1500	3000
	search radius	NA	NA	15	15
	min overlap	NA	NA	0.5	0.5
	allowed slice jumps	NA	NA	2	2
**Post-processing**	delete flat structures	NA	NA	yes	yes
	finalise	NA	NA	yes	yes
	split type	NA	NA	None	None

In all those situations Nessys generated results with reasonably high accuracy (Fig S9)

E. Additional analysis of Nessys outputs

E.1. Tcf15 expression analysis

The Tcf15 expression analysis shown in Fig 5 was performed in PickCells. The Nessys segmentation module was used to perform the segmentation of the full E8.75 image of the DISCEPTS dataset. For Fig 5C and D, embryonic regions were manually annotated using the Nessys 3D painter module. A rule was created in PickCells to assign nuclei to a given embryonic region if their shape overlapped with the annotated region by at least 95%. Fig 5A and B 3D representations were created using the 3D scene module in PickCells.

E.2. Cell tracking and Neighbour exchange analysis

The tracking results presented in Fig 7 were obtained with the tracking module of PickCells (https://framagit.org/pickcellslab/pickcells-essentials/tree/develop/pickcells-tracking) using manually edited Nessys segmentation as input.

E.2.1. Neighbour exchange rate definition

First, the neighbour graph was computed as described in (section B3 ‘Neighbour Difference’) for each time point. Then, we compared each nucleus to itself in the previous time point to obtain the number of unique neighbours that have been lost (N_{loss} = set difference of neighbours at t and neighbours at t+1) and the number of unique neighbours that have been gained (N_{gain} = set difference of neighbours at t+1 and neighbours at t) during one time frame:

We then defined the neighbour exchange rate (NER) for a given branch as follows:

$$NER = \frac{\sum_{t=t_{start}}^{t_{end}} Nloss_t + \sum Ngain_t}{t_{end} - t_{start}}$$

where t_{start} and t_{end} are the time frame of the first and last node of the branch respectively.

E.2.2. Nuclei and Branches classification

Sox1 classification: We defined a branch as Sox1+ if the mean of average Sox1 intensity of nuclei composing the branch was above a given threshold. This threshold was set subjectively based on both the shape of the distribution of nuclei mean intensities and based on visual inspection of the movie (Movie S3 – PickCells allows us to click on individual nuclei or branches to obtain the features values of the selected object).

Dividing nuclei: Nuclei were defined as ‘dividing’ based on their position in the lineage tree, i.e a nucleus with two outgoing links and its depth one neighbouring nuclei in the tree were defined as ‘dividing’ (Fig 7G).

Apoptotic nuclei: apoptotic nuclei were defined as the leaf nodes in a tree which did not reach the end of the movie.

Dividing branch: A branch for which the last node corresponds to a dividing nucleus.

Apoptotic branch: A branch for which the last node corresponds to an apoptotic nucleus.

Surviving Branch: A branch for which the last node was neither a dividing nor an apoptotic nucleus.

Above/ below plane classification: We defined a lineage as dividing above the epithelial plane if the average Z coordinates of dividing nuclei was above 6.5 µm which corresponded to the 3rd quartile plus 1.5 times the inter-quartile range of the distribution of Z coordinates of non dividing nuclei (Fig 7G). Branches were then defined as above or below based on the category of the tree they belonged to. Lineages with no divisions were excluded.

1. Pal S, Bhowmick P. Estimation of discrete curvature based on chain-code pairing and digital straightness. 2009 16th IEEE International Conference on Image Processing (ICIP). 2009. pp. 1097–1100. doi:10.1109/ICIP.2009.5413475

2. R Core Team. R: A Language and Environment for Statistical Computing [Internet]. Vienna, Austria: R Foundation for Statistical Computing; 2013. Available: http://www.R-project.org/

3. Ljosa V, Sokolnicki KL, Carpenter AE. Annotated high-throughput microscopy image sets for validation. Nature Methods. 2012;9: 637. doi:10.1038/nmeth.2083

4. Stegmaier J, Amat F, Lemon WC, McDole K, Wan Y, Teodoro G, et al. Real-Time Three-Dimensional Cell Segmentation in Large-Scale Microscopy Data of Developing Embryos. Developmental Cell. 2016;36: 225–240. doi:10.1016/j.devcel.2015.12.028
